# Conjunctival Sac‐Derived *Lactobacillus mucosae* MM‐1 Metabolite Indole‐3‐Lactic Acid Attenuates Lens‐Induced Myopia in Mice via AHR Activation and TGF‐β/Smad Inhibition

**DOI:** 10.1111/1751-7915.70426

**Published:** 2026-08-07

**Authors:** Yanqing Li, Qiuyu Wu, Xilong Zhang, Luchuan Liu, Leiping Ding, Jingjing Xu, Chiwen Cheng, Yangyang Peng, Kang Yu, Wen Yao, Yijie Pi, Jiale Li, Di Wu, Jing Wei, Yifeng Yu, Tingtao Chen

**Affiliations:** ^1^ Department of Ophthalmic Center, The Second Affiliated Hospital, Jiangxi Medical College Nanchang University Nanchang Jiangxi Province China; ^2^ School of Pharmacy, Jiangxi Medical College Nanchang University Nanchang Jiangxi Province China; ^3^ Jiangxi Province Key Laboratory of Bioengineering Drugs, Institute of Translational Medicine, Jiangxi Medical College Nanchang University Nanchang China; ^4^ Queen Mary School, Jiangxi Medical College Nanchang University Nanchang Jiangxi Province China

**Keywords:** AHR, high myopia, Indole‐3‐lactic acid, *Lactobacillus mucosae*, TGF‐β/Smad signalling pathway

## Abstract

Emerging research has revealed a diverse microbiota in the high myopia (HM) conjunctival sac, highlighting the therapeutic potential of modulating this community to influence disease outcomes. However, current research on targeted modulation of this microbiota for myopia intervention lacks in‐depth investigation and definitive conclusions regarding its efficacy and underlying mechanisms. In this study, high‐throughput sequencing and culturomics were first used to screen the conjunctival sac microbiota of healthy people, identifying *Lactobacillus mucosae* MM‐1 as a potential probiotic for myopia control. Then, animal studies further confirmed that *L. mucosae* MM‐1 significantly attenuated lens‐induced myopia, improved COL1A1 expression and scleral integrity and elevated *Lactobacillus* abundance. Furthermore, untargeted metabolomics analysis suggested that indole‐3‐lactic acid (ILA) may be a key substance for *L. mucosae* MM‐1 to exert its effects for myopia control. Mechanistically, pharmacological ILA supplementation supports the involvement of AHR in TGF‐β/Smad pathway inhibition, accompanied by altered COL1A1 and α‐SMA expression and attenuation of lens‐induced myopia in mice. Therefore, these findings suggest that *L. mucosae* MM‐1 may attenuate lens‐induced myopia in mice, at least in part, through ILA‐mediated regulation of the TGF‐β/Smad pathway, and offer a theoretical foundation for future therapeutic strategies based on conjunctival sac microbiome manipulation.

## Graphical abstract

This study proposes a ‘conjunctival sac microecology‐metabolite‐scleral remodelling’ regulatory axis, highlighting the potential role of conjunctival sac microbiota in maintaining ocular surface health and regulating lens‐induced myopia in mice, providing an important theoretical foundation for the development of microbiome‐based interventions for myopia control.

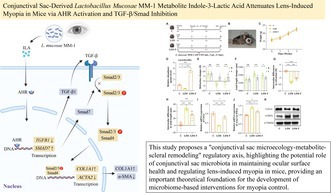

## 1 Introduction

High myopia (HM) poses a grave and growing threat to global vision, driven by genetic and environmental factors that precipitate devastating complications like macular degeneration, retinal detachment, glaucoma and cataract, culminating in irreversible vision loss and exacting a heavy societal toll (Haarman et al. 2020). Currently, optical correction and refractive surgery are widely employed for myopia management. However, optical correction often involves daily inconveniences, while surgical interventions entail the risk of infection and dry eye syndrome, which significantly restrict their broader clinical application and long‐term patient experience. Therefore, identifying risk factors associated with HM and developing more effective prevention and control strategies are of critical clinical importance.

The ocular surface is primarily composed of the cornea and conjunctiva, colonised by a variety of microorganisms that play a crucial role in local immune development, host metabolic regulation and pathogen resistance (Aristoteli et al. 2003; Berry 2002; Li et al. 2020). In a clinical trial, Zhao et al. identified conjunctival sac microbiota dysbiosis in patients with HM compared to healthy individuals via 16S rRNA sequencing, revealing a significant association between reduced *Lactobacillus vini* abundance and axial length (AL) elongation (Zhao et al. 2025). Notably, Iovieno et al. reported that *L. acidophilus* eye drops administered over 4 weeks significantly improved the clinical symptoms and signs of active mild to moderate vernal keratoconjunctivitis (VKC) (Iovieno et al. 2008). Animal studies demonstrated that *L. vini* eye drops downregulated the ocular surface inflammatory markers IL‐6 and TNF‐α, inhibited scleral remodelling and reduced myopic phenotypic changes without affecting corneal morphology (Zhao et al. 2025). Therefore, the conjunctival sac microbiota may serve as both a potential biomarker for assessing the development and severity of HM and a target for intervention.

Microbiota dysbiosis‐related diseases comprise a group of disorders whose onset and/or progression is associated with dysbiosis and that may be ameliorated through microbiota reconstruction (Zhang, Pi, et al. 2026). Probiotics are defined as live, non‐pathogenic microorganisms that confer health benefits on the host when administered in adequate amounts (Reid et al. 2003). Lactic acid bacteria (LAB), as one of the most widely applied and extensively studied groups among probiotics, naturally colonise the oral cavity, skin, gastrointestinal tract, vagina and ocular surface (Zilliox et al. 2020). *Lactobacillus mucosae* , a prominent member of LAB, alleviates colonic inflammation by downregulating the expression of IL‐1β, TNF‐α and pSTAT3, modulating the gut microbiota, and increasing indole‐3‐acetic acid (IAA) levels (Sun et al. 2024; Zhang et al. 2022). Animal studies demonstrated that tryptophan metabolites from Lactobacilli activated the aryl hydrocarbon receptor (AHR), suppressed the TGF‐β/Smad signalling pathway, ameliorated atherosclerosis, enhanced intestinal barrier function and alleviated colonic inflammation (Jing et al. 2024; Luo et al. 2024). Moreover, the tryptophan metabolite 3‐IAA was demonstrated to promote COL1A1 protein expression in sclera and delay HM progression (Li et al. 2024). However, whether *L. mucosae* can improve HM and the specific pathways involved remain to be elucidated.

In this study, we conducted a comprehensive microbiome analysis of conjunctival sac samples from patients with HM to investigate the clinically relevant relationship between conjunctival sac microbiota and HM. Additionally, we employed a −30 D lens‐induced HM animal model and an in vitro hypoxia cell model to explore the underlying mechanisms. We found that *L. mucosae* MM‐1 exerts a beneficial effect on delaying HM progression by regulating scleral extracellular matrix (ECM) remodelling and enhancing COL1A1 expression, thereby retarding the development of refractive error and AL. Metabolomic profiling and ELISA quantification identified ILA as a key metabolite produced by *L. mucosae* MM‐1. Pharmacological supplementation with ILA supports the involvement of AHR in TGF‐β/Smad pathway inhibition, accompanied by increased COL1A1 expression and improved ECM remodelling. These results extend previous observations by identifying *L. mucosae* MM‐1 as a conjunctival sac‐derived probiotic that may attenuate lens‐induced myopia in mice through ILA/AHR/TGF‐β signalling and by demonstrating that exogenous ILA administration via the conjunctival sac serves as a pharmacological proof‐of‐concept for this mechanism. This offers a theoretical foundation for developing microbiota‐based strategies for myopia prevention and control, with significant theoretical and clinical implications.

## 2 Method Details

### 2.1 Research Subjects

The study was conducted in Nanchang City, Jiangxi Province, China, and recruited patients attending the ophthalmology outpatient clinic at the Second Affiliated Hospital of Nanchang University. All participants were selected according to inclusion and exclusion criteria (Chen et al. 2023, 2024; Kappel et al. 2020). Inclusion criteria: (1) According to the quantitative standards of the International Myopia Institute (IMI), SE was defined as spherical power plus 1/2 cylindrical power. HM is defined as SE ≤ −6.00 D; Healthy control (HC) group: −0.5 ≤ SE < 0.5 D (Flitcroft et al. 2019); (2) Informed consent with understanding of study details; (3) Willingness to undergo ophthalmic examination and specimen collection. Exclusion criteria: (1) Eyelid disorders: trauma, entropion, or lagophthalmos; (2) Conjunctival disorders: infectious conjunctivitis, allergic conjunctivitis, pterygium, or conjunctival scarring; (3) History of severe ocular chemical injury or trauma; (4) History of ocular surgery or contact lens wear within the past 3 months; (5) History of systemic infection within the past month; (6) History of antibiotic or probiotic use (oral or topical) within the past month; (7) Pregnancy or presence of systemic disease; (8) History of hepatitis or neoplastic disease; (9) Tobacco use or alcohol abuse; (10) Other conditions incompatible with this study.

The study employed micro‐efficiency for sample size estimation, which is an efficiency and paired sample size estimator based on the application of Permutational Multivariate Analysis of Aariance (PERMANOVA) (Kelly et al. 2015). The sample size was determined with reference to a previous ocular microbiome study that utilised a low‐abundance 16S rRNA dataset, setting a minimum of 30 samples to achieve a statistical power of 0.8 at a significance level (α) of 0.05 (Baim et al. 2018). This study intends to enrol 30 participants in each of the HM and HC groups to meet the research requirements.

The study strictly adheres to the Declaration of Helsinki and the Biosafety Law of the People's Republic of China. The research protocol has been approved by the Biomedical Research Ethics Committee of the Second Affiliated Hospital of Nanchang University (Approval No.: O‐Yiyan Lun Shen [2025] No. (20)). All participants were fully informed of the study's purpose, procedures and potential risks, and signed informed consent forms.

### 2.2 Collection of Medical History

Baseline data were systematically collected for all participants, including demographic characteristics (sex, age, height, weight and education level), lifestyle habits (smoking and alcohol consumption history) and clinical history (past diseases, surgical or trauma history, medication use and allergies). Educational qualifications are categorised as follows: (a) Primary school, (b) Junior high school, (c) Senior high school.

### 2.3 Standard Ophthalmic Evaluation

All participants underwent a comprehensive ophthalmic examination. All participants received Uncorrected Visual Acuity (UCVA) testing, measurement of bilateral spherical refractive error using an automated refractometer (ARK‐1 s, Nidec Corporation, Japan), subjective refraction and cycloplegic refraction to determine BCVA, slit‐lamp examination (SL 115 Classic, Carl Zeiss AG, Germany) and dilated fundus examination to assess the anterior segment and fundus, non‐contact tonometer (NT‐510, Nidek Co. Ltd., Japan) to measure IOP in both eyes to exclude high IOP and glaucoma and IOL‐Master (IOLMaster 700, Carl Zeiss, Germany) to obtain AL data.

### 2.4 OSDI Questionnaire Assessment

Given that clinical HM patients are more prone to ocular discomfort such as dry eye, all participants were required to complete the OSDI questionnaire to assess subjective dry eye symptoms. The OSDI questionnaire is a reliable and valid assessment tool for evaluating subjective symptoms of ocular surface disease and their impact on visual function. It measures the presence and duration of ocular discomfort over the preceding 3 months using a severity scale of 0–4 (0 = never experienced; 1 = occasionally; 2 = half the time; 3 = most of the time; 4 = all the time). The final OSDI score = A*25/B, where A is the total sum of scores for all questions and B is the total number of questions. The OSDI score ranges from 0 to 100, with higher scores indicating greater severity (Michel et al. 2009; Schiffman 2000).

### 2.5 Conjunctival Sac Microbiota Sample Collection

All samples were collected by the same person at a fixed location. Collect ocular surface specimens using single‐use sterile collection swabs, strictly adhering to aseptic technique to ensure sample integrity. Specimen collection shall be conducted in a suitable examination room providing adequate lighting, temperature and humidity for obtaining conjunctival swabs from right eyes. The sampling procedure shall be performed as follows: all operations must be conducted under strict aseptic conditions. Gently wipe the patient's eyelid skin twice with a saline‐soaked cotton swab, then proceed to sampling using a conjunctival swab. During sampling, avoid contact between the swab and the eyelid or eyelashes, retaining the swab head as the specimen. Subsequently, promptly place the specimen into a sterile Eppendorf (EP) tube containing DNA preservation solution and store at −80°C for future use.

### 2.6 16S rRNA Gene Amplicon Sequencing and Contamination Control

Library preparation and sequencing were performed by Shanghai Personal Biotechnology Co. Ltd. Given the low‐biomass nature of conjunctival sac samples, stringent contamination controls were integrated into the experimental and computational pipelines. For each 96‐well plate of DNA extraction, an extraction blank (nuclease‐free water) was included and processed in parallel with the samples; a no‐template control was also incorporated during PCR amplification, and a sequencing blank was added in each run to monitor reagent and cross‐contamination. Samples from HM and HC groups were randomly assigned to extraction batches and sequencing lanes to avoid systematic batch effects. Raw reads were processed using the DADA2 pipeline, including primer removal, quality filtering, denoising, paired‐end merging and chimera removal, which yielded ASVs. Putative contaminants were identified and filtered using the R package decontam (version 1.18.0), applying the frequency method to extraction blanks and the prevalence method to no‐template controls with the default threshold. After contaminant removal, samples with fewer than 500 total reads were excluded from downstream analysis. Taxonomy was assigned to the retained ASVs using the Greengenes database (Release 13.8) (DeSantis et al. 2006), and an ASV abundance matrix was constructed for subsequent community composition analysis, alpha diversity estimation and functional prediction.

### 2.7 Acquisition and Identification of HSF

Human primary scleral fibroblasts (HSF, Catalogue No. BNCC363004) were obtained from BeNa Culture Collection (BNCC, Suzhou, China) on April 23, 2025. The cells were derived from the scleral tissue of a male human donor. No RRID is available for this primary cell line. For authentication, standard STR profiling against cell line databases is not applicable for these finite‐lifespan primary cells; instead, species and tissue origin were certified by the supplier, and the fibroblast phenotype was confirmed by Vimentin immunofluorescence staining, with > 90% of cells exhibiting strong positivity. This primary cell line is not listed in the ICLAC Register of Misidentified Cell Lines. All functional experiments were performed using cells at passages P2–P5. Routine mycoplasma testing was conducted using a PCR‐based detection kit (Catalogue No. CA1081, Solarbio, Beijing, China) prior to all experiments, and all batches were confirmed negative (last tested on May 30, 2025).

### 2.8 Cell Hypoxia Model

HSF cells were cultured using the appropriate HSF cell culture medium in an incubator maintained at 37°C, 5% CO_2_, and saturated humidity. Cells were digested and passaged using 0.25% trypsin (Solarbio, Beijing, China), with the third‐passage cells employed for experiments. Subsequently, cells were placed in a tri‐gas hypoxic incubator at 5% O_2_ for 24 h to induce the hypoxic HSF model. Normal oxygenation (21% O_2_) served as the control (Lin et al. 2024; Pan et al. 2021; Xue et al. 2024).

### 2.9 Probiotic Cultivation and Screening

Conjunctival sac swabs collected from the healthy control (HC) group were placed into sterile EP tubes, followed by vortexing, plate coating and single‐colony selection and identification. This process resulted in the identification of two probiotic strains belonging to the genus *Lactobacillus*: *L. plantarum* and *L. mucosae* . The strains were cultured on MRS medium in a 37°C aerobic incubator. They were co‐incubated with the hypoxia HSF model for 4 h, after which changes in cell migration and viability were assessed via cell scratch assays and CCK‐8 assays.

### 2.10 Evaluation of Probiotic Properties in Vitro

1. Bacterial staining: Spread the bacterial suspension into a thin layer on a microscope slide. Once the suspension has air‐dried, pass it rapidly through a flame several times (heat fixation). Add an appropriate amount of crystal violet stain, then gently rinse away excess dye under running water. After the slide has dried, observe the morphology of the bacteria under a microscope using an oil immersion objective.
2. Growth curve: Take MRS liquid medium and incubate overnight to activate probiotics. The following day, add 1 mL of bacterial culture to 200 mL of MRS liquid medium and incubate in a 37°C aerobic incubator. From the time of inoculation, measure the optical density (OD) at 600 nm every 2 h, recording continuously for 48 h. Perform dilution plate spreading and counting at each time point. Plot a growth curve with time as the x‐axis and with OD and viable cell count (CFU/mL) on the primary and secondary y‐axes, respectively.
3. Bacteriostatic test: Common ocular pathogens were revived in LB liquid medium: *S. epidermidis* , *E. coli* , *S. aureus* , *P. mirabilis* and *P. aeruginosa* . Apply 30 μL of each bacterial suspension onto the surface of LB agar plates. Each group comprised three experimental groups (probiotic bacterial suspensions) and one blank control group (liquid culture medium). After incubation at 37°C for 8 h, the diameter of the inhibition zones on each plate was observed and recorded.
4. Adhesion capacity: In a 6‐well plate, human corneal epithelial cells were seeded onto coverslips. Once cell coverage reached 30%, complete culture medium and bacterial suspension were added for co‐culture for 2 h. Subsequently, the samples were fixed with methanol, stained with crystal violet, observed under a microscope and photographed.
5. Antimicrobial resistance testing: Antimicrobial susceptibility discs were used, specifically including tetracycline (TET), trimethoprim/sulfamethoxazole (SXT), penicillin (PEN), lincomycin (MY), gentamicin (GEN), erythromycin (ERY/E), ceftriaxone (CTR), ciprofloxacin (CIP), chloramphenicol (C) and ampicillin (AMP). Each was placed on the surface of agar plates pre‐coated with probiotic bacterial suspension. Incubated at 37°C for 16–18 h, the diameter of the inhibition zone was measured and recorded.
6. Haemolysis test: Inoculate probiotics and *S. aureus* separately onto the surface of BHI agar plates containing sheep blood. Incubate at 37°C for 24 h, then observe and record any haemolysis phenomena.
7. Antioxidant capacity assay: Centrifuge the activated probiotics, collect the supernatant and refrigerate for subsequent use. Measure the scavenging capacity against DPPH radicals, hydroxyl radicals (‐OH), superoxide radicals (O_2_^−^), chelation capacity towards Fe^2+^ and total reducing power.
8. Centrifugation performance: Centrifuge the probiotic culture suspension at 5000–7000 g for 10 min. Measure the OD_600_ value of the supernatant, calculate the wet weight of bacterial sludge per unit volume of culture (sludge weight/culture volume) and determine the sludge concentration factor (fermentation broth weight/sludge weight).

### 2.11 Solid–Liquid Separation of Bacterial Cultures

Centrifuge the bacterial suspension (4°C, 10,000 × g, 15 min), collecting the supernatant and bacterial pellet separately. Immediately filter the collected supernatant through a 0.22 μm sterile filter membrane to remove bacteria, then aliquot. Store the bacterial pellet at −80°C for future use. To preserve surface antigen integrity while achieving effective inactivation, the bacterial pellet was irradiated under ultraviolet light (wavelength 254 nm) for 15 min. For the bacterial supernatant, the conditioned medium was diluted with fresh MRS medium to final concentrations of 10%, 20%, 30% and 40% (v/v), and HSF cells were then treated with these dilutions. For the UV‐inactivated bacterial cells, the bacterial pellets were washed twice with sterile PBS to remove residual culture medium components, resuspended in fresh MRS medium, and then co‐incubated with HSF cells at varying cell‐to‐bacteria ratios: 1:1000, 1:100, 1:10, 1:1 and 10:1 (cells: bacteria).

### 2.12 Construction and Evaluation of HM Animal Models

Four‐week‐old male C57BL/6 mice were procured from Henan Sike Beisi Biotechnology Co. Ltd. (Animal Licence No.: SCXK (Yu) 2020‐0005) and housed in an SPF environment. Prior to experimentation, ophthalmoscopic and refraction examinations were conducted to exclude mice with significant anisometropia. An HM mouse model of LIM was established by fitting experimental mice with −30.0 D lenses. The device is suspended above the eye, with ample gaps on the sides to ensure normal air circulation. The lenses were positioned centrally over the pupils without impeding blinking or other activities. Daily inspections were conducted to adjust lens placement as required, ensuring lenses were promptly cleaned or replaced to maintain normal feeding and drinking behaviour. Measures such as clipping claws or wrapping toes were employed to minimise lens damage from scratching. During the experiment, regular examinations of both eyes in mice were conducted to exclude those exhibiting other ocular pathologies or ocular scratches. A minimum of 6.00 D myopic shift in the right eye relative to the left eye was considered indicative of successful induction of the high myopia model. During the study, both eyes were imaged using a small animal magnetic resonance imaging system (Aspect Imaging, Israel), with AL measured via ImageJ (version 1.54). Retinoscopy was performed with a streak retinoscope (Suzhou 66Vision Technology Co. Ltd., China).

The study comprised three rounds of animal experiments. The first round of animal experiments was grouped as follows: Control group (C, *n* = 10), fitted with 0 D lenses and administered PBS topically to the ocular surface three times daily. Lens‐induced myopia model group (LIM, *n* = 10), fitted with −30 D lenses and administered PBS topically to the ocular surface three times daily. Lens‐induced myopia treatment group (LIM‐T, *n* = 10), fitted with −30 D lenses and administered probiotic solution topically to the ocular surface three times daily (5 μL, 1 × 10^6^ CFU/mL) (Zhao et al. 2025). The probiotic eye drops are prepared from a plateau‐phase bacterial culture, following centrifugation, bacterial collection and resuspension in PBS. The intervention frequency was determined based on the clinical administration frequency of eye drops to ensure their full efficacy on the ocular surface. Second round of animal experiments grouping: Model group (M, *n* = 10): fitted with −30 D lenses and administered 5 μL of 0.05% DMSO topically to the ocular surface three times daily. Metabolite treatment group (MT, *n* = 10): fitted with −30 D lenses and administered 5 μL of 0.5 mM ILA in 0.05% DMSO topically to the ocular surface three times daily. Metabolite and inhibitor treatment group (MTI, *n* = 10): fitted with −30 D lenses and administered 5 μL of the mixture (0.5 mM ILA and 10 μM CH‐223191 in 0.05% DMSO) topically to the ocular surface three times daily. Third round of animal experiments grouping: M: Model group (*n* = 10), fitted with −30 D lenses and administered 5 μL of 0.05% DMSO topically to the ocular surface three times daily. LT: *L. mucosae* MM‐1 treatment group (*n* = 10), fitted with −30 D lenses and administered probiotic solution topically to the ocular surface three times daily. LTI: *L. mucosae* MM‐1 and inhibitor treatment group (*n* = 10), fitted with −30 D lenses and administered 5 μL of CH‐223191 (10 μM) followed by 5 μL of probiotic solution (10^6^ CFU/mL), both applied topically to the ocular surface three times daily. This study has been approved by the Nanchang University Laboratory Animal Welfare Ethics Committee (Approval No.: NCULAE‐20221031187).

### 2.13 Histopathological Changes

Mice were euthanised, and eyeballs were fixed in 4% paraformaldehyde for 24 h. Standard procedures for dehydration, clearing and paraffin embedding were followed. Trimmed paraffin blocks were sectioned into continuous slices 3–5 μm thick. Sections were stained using a HE staining kit according to the manufacturer's instructions. Scleral thickness and interlamellar variations were observed and photographed using an Olympus microscope (Olympus Corporation, Japan).

### 2.14 Reverse Transcription qPCR (RT‐qPCR) for Gene Expression Analysis

Total RNA was extracted using a high‐purity RNA extraction kit (TransZol Up Plus RNA Kit). For the in vivo study, scleral tissues were meticulously dissected from mouse eyes. For the in vitro study, HSF cells were lysed after culture and treatment. Total RNA reverse transcription was performed using the Hifair AdvanceFast One‐step RT‐gDNA Digestion SuperMix for qPCR kit. The RT‐qPCR reaction system was prepared using the Hieff qPCR SYBR Green Master Mix (No Rox) kit. Real‐time fluorescent quantitative PCR (RT‐qPCR) experiments were conducted using the Gentier 96R fully automated PCR analysis system (TianLong). Following reaction completion, data analysis was performed with GAPDH serving as the standard control. Target gene quantification was achieved via the ΔΔCt method (comparative Ct method). Primer sequences are detailed in Table S1.

### 2.15 Quantitative PCR (qPCR) for Microbial Abundance Analysis

Bacterial DNA was extracted from conjunctival sac secretions using the QIAamp DNA Microbiome Kit (Qiagen, Germany). DNA concentration and purity were measured with a NanoDrop 2000 spectrophotometer, and the DNA was stored at −20°C until further analysis. qPCR was performed on a Gentier 96R fully automated PCR analysis system (TianLong). The primers used for the detection of the *Lactobacillus* genus were: forward, 5′‐TGGAAACAGRTGCTAATACCG‐3′ and reverse, 5′‐GTCCATTGTGGAAGATTCCC‐3′. The primers targeting the conserved region of the bacterial 16S rRNA gene (serving as a reference for total bacterial load) were: forward, 5′‐ACTCCTACGGGAGGCAGCAGT‐3′ and reverse, 5′‐TATTACCGCGGCTGCTGGC‐3′ (Wang et al. 2024).

### 2.16 Western Blot Analysis

Total proteins were extracted using RIPA tissue/cell lysis buffer (Solarbio, Beijing, China) supplemented with protease inhibitors. For the in vivo study, proteins were extracted from meticulously dissected mouse scleral tissues. For the in vitro study, HSF cells were lysed directly in the buffer after treatment. Add SDS‐PAGE loading buffer (5×) (Solarbio, Beijing, China) equivalent to one‐quarter of the sample volume, mix thoroughly by shaking, boil at 100°C for 10 min, and store at −80°C. Experimental procedures comprised gel preparation, electrophoresis, membrane transfer, blocking, primary antibody incubation, secondary antibody incubation and development. Band intensity was quantified using ImageJ (version 1.54). Antibody usage details are provided in Table S2.

### 2.17 Metabolomic Analysis

Probiotic supernatant samples were submitted to Shanghai Paisenuo Biotechnology Co. Ltd. for testing. The samples underwent quality control, metabolite extraction, instrument analysis and data analysis. Non‐targeted metabolomic analysis was performed using Ultra‐Performance Liquid Chromatography‐Quadrupole‐Time‐of‐Flight‐Tandem Mass Spectrometry (UPLC‐Q‐TOF‐MS/MS). The acquired data underwent PCA, Partial Least Squares Discriminant Analysis (PLS‐DA), OPLS‐DA, univariate statistical analysis (volcano plots), identification of significantly different metabolites and clustering analysis of differential metabolites. This approach enabled the identification of metabolites exhibiting differential expression. For experimental purposes, ILA (Cat. No. HY‐113099, MedChemExpress) and 3‐IAA (Cat. No. HY‐18569, MedChemExpress) were administered at a concentration of 0.5 mM in relevant cellular assays to evaluate their efficacy (Zhang et al. 2023, 2026).

### 2.18 Cell Scratch Assay

Using a marker pen and ruler, draw several evenly spaced horizontal lines on the back of a 6‐well plate. Seed cells overnight to achieve a 90%–100% confluence rate. Scratch the cells vertically along the lines using a 200 μL pipette tip. Wash with PBS and add serum‐free medium for continued culture. Observe the scratches under an inverted microscope at designated time points (0, 24, 48 h) and document photographs. The mean intercellular scratch area was analysed using ImageJ (version 1.54) software to assess cell migration capacity.

### 2.19 CCK‐8 Assay

Cells were diluted and seeded into a 96‐well plate, followed by incubation at 37°C until they reached confluence. After 48 h, the old medium was aspirated. A CCK‐8 reaction solution (containing 10% CCK‐8 from Beyotime Biotechnology in complete medium) was prepared in the dark, and 100 μL of this solution was added to each well. The plate was then incubated in the dark at 37°C for 2 h. Subsequently, the absorbance (OD value) of each well was measured at 450 nm using an absorbance microplate reader (CMAX PLUS, Molecular Devices).

### 2.20 Transwell Assay

Dilute the cell suspension to 2.5 × 10^5^ cells/mL, with each chamber intended for inoculation with 5 × 10^4^ cells. Inoculate the upper chamber with 200 μL of cell suspension, and fill the lower chamber with 500 μL of complete medium containing 10% foetal bovine serum (FBS). Incubate in a 37°C incubator. After 24 h, add 4% paraformaldehyde to each well for 15 min, then wash with PBS. Add 0.1% crystal violet solution to each well for staining, wash with PBS for 10 min, then air‐dry. Carefully remove non‐migrated cells and excess crystal violet from the Transwell chamber using a moistened cotton swab. Cover with a coverslip and observe under a microscope, counting the number of cells in each field of view to assess cellular migration or invasion capacity.

### 2.21 Cell Viability Assay

Cell viability was assessed using the Calcein‐AM/PI (Beyotime Biotechnology) dual‐fluorescent staining method following intervention. Cells were seeded into 96‐well plates and cultured overnight to allow attachment before being divided into treatment groups. Culture medium was aspirated, wells were washed with PBS, and 100 μL of freshly prepared staining working solution was added to each well. The plates were then incubated in the dark at 37°C for 30 min. Following the incubation, the samples were immediately observed and imaged under a fluorescence microscope (Leica ICC50 W, Leica Microsystems GmbH, Germany). Viable cells, stained with Calcein‐AM, exhibited green fluorescence (Ex/Em = 494/517 nm), whereas dead cells, stained with PI, showed red fluorescence (Ex/Em = 535/617 nm). Images were captured from randomly selected fields for subsequent counting.

### 2.22 Enzyme‐Linked Immunosorbent Assay (ELISA)

Scleral tissue samples were homogenised in 100 μL of ice‐cold phosphate‐buffered saline (PBS) containing protease inhibitors using a tissue grinder, followed by ultrasonication. The homogenates were centrifuged at 10,000 × g for 15 min at 4°C to remove cellular debris and impurities. The supernatant was carefully collected for subsequent analysis. The concentration of ILA was measured using a commercial ELISA kit (Jianglai Bio, China) according to the manufacturer's instructions. Standards were prepared as directed. The assay procedure included sample/standard addition, incubation, washing, addition of horseradish peroxidase (HRP)‐conjugated secondary antibody, another washing step, substrate incubation for colour development, and termination of the reaction. Absorbance was measured at 450 nm using a microplate reader.

### 2.23 AHR Inhibitor Preparation

The AHR inhibitor CH223191 (CAS No. 301326‐22‐7) was purchased from MedChemExpress (MCE). For experimental use, a working solution of 10 μM was prepared by diluting the compound in culture medium containing 0.05% dimethyl sulfoxide (DMSO) immediately prior to the assays (Zhang et al. 2023).

### 2.24 Statistical Analysis

Statistical analysis was conducted using SPSS version 23.0 (SPSS Inc., Chicago, IL, USA), GraphPad Prism version 9 (GraphPad Software, San Diego, CA, USA) and R software (v3.2.1). The normality of continuous variables was assessed using the Shapiro–Wilk test. Data are presented as mean ± standard deviation (SD) for normally distributed variables, and as median (interquartile range) for non‐normally distributed variables. Categorical variables are described as frequency (percentage) and were compared using the chi‐square test. For intergroup comparisons of normally distributed continuous variables, one‐way analysis of variance (ANOVA) followed by Tukey's post hoc test was used for multiple comparisons, while the independent samples *t*‐test was used for two‐group comparisons. For non‐normally distributed variables, the Mann–Whitney *U* test was applied. For body weight data, comparisons among the three groups were performed at each individual time point (Weeks 0–4) using one‐way ANOVA followed by Tukey's post hoc test. For paired interocular data (left vs. right eyes for refractive error and axial length), two‐way ANOVA with Šidák's multiple comparisons test was applied to account for the paired structure. For microbiome and metabolomics data, multiple testing was corrected using the false discovery rate (FDR) method, with a significance threshold of *q* < 0.05. Effect sizes and 95% confidence intervals were calculated where applicable. Sensitivity analyses were performed by excluding outliers (defined as values beyond ±2 SD from the mean) to confirm the robustness of the results. Spearman's correlation analysis was employed to evaluate the correlation between myopia‐related indicators and key species of the conjunctival sac microbiota. All statistical tests were two‐tailed, with significance defined as *p < 0.05, **p < 0.01, ***p < 0.001. Specific statistical details for each experiment are provided in their respective figure legends. It should be noted that researchers were not blinded during grouping and experimental procedures.

## 3 Result

### 3.1 Differences in the Conjunctival Sac *Lactobacillus* May be a Potential Factor of HM

To investigate the role of the conjunctival sac microbiota in the progression of HM, 30 HM patients (HM group) and 30 healthy volunteers (HC group) were enrolled (Figure S1). As shown in Table 1, while there were no significant differences between the 2 groups in sex, age, body mass index (BMI), white‐to‐white (WTW), intraocular pressure (IOP), Ocular Surface Disease Index (OSDI) score, or education level, there were significant differences in HM‐related clinical parameters, including spherical equivalent (SE), AL, anterior chamber depth (ACD) and lens thickness (LT) (*p <* 0.05).

**TABLE 1:** Main baseline characteristics of HC and HM.

Characteristic	Group		*p*
	HC, *N* = 30	HM, *N* = 30	
Sex, *n* (%)			0.793^a^
Female	12 (40.0%)	13 (43.3%)	
Male	18 (60.0%)	17 (56.7%)	
Age, year	14.00 (12.00, 16.00)	15.00 (13.00, 17.00)	0.106^b^
BMI, kg/m^2^	22.09 (20.28, 23.12)	21.27 (20.45, 21.80)	0.348^b^
SE‐OD, D	−0.3 (−0.3, −0.3)	−9.1 (−10.0, −8.0)	< 0.001^b^
SE‐OS, D	−0.3 (−0.3, −0.1)	−9.8 (−10.5, −8.8)	< 0.001^b^
AL‐OD, mm	23.91 (23.61, 24.07)	28.10 (27.11, 28.84)	< 0.001^b^
AL‐OS, mm	23.77 ± 0.17	28.65 ± 0.86	< 0.001^c^
ACD‐OD, mm	3.13 ± 0.18	3.30 ± 0.21	0.002^c^
ACD‐OS, mm	3.15 (2.95, 3.24)	3.42 (3.25, 3.53)	< 0.001^b^
LT‐OD, mm	4.02 (3.83, 4.21)	3.39 (3.28, 3.61)	< 0.001^b^
LT‐OS, mm	4.01 (3.81, 4.21)	3.53 (3.29, 3.68)	< 0.001^b^
WTW‐OD, mm	11.80 (11.70, 11.90)	11.80 (11.70, 11.90)	0.446^b^
WTW‐OS, mm	11.82 ± 0.14	11.81 ± 0.16	0.660^c^
OSDI	4.50 (3.00, 6.00)	5.00 (3.00, 7.00)	0.301^b^
IOP‐OD, mmHg	15.21 ± 1.92	15.42 ± 1.94	0.666^c^
IOP‐OS, mmHg	16.00 (14.00, 17.00)	15.50 (14.00, 17.00)	0.690^b^
Educational, *n* (%)			0.422^a^
a	11 (36.7%)	11 (36.7%)	
b	9 (30.0%)	13 (43.3%)	
c	10 (33.3%)	6 (20.0%)	

*Note:* Data are presented as mean ± standard deviation (SD), median with interquartile range (IQR), or *n* (%).

Abbreviations: ACD, anterior chamber depth; AL, axial length; BMI, body mass index; HC, healthy control; HM, high myopia; IOP, intraocular pressure; LT, lens thickness; OD, right eye; OS, left eye; OSDI, ocular surface disease index; SE, spherical equivalent; WTW, white‐to‐white.

^a^ Pearson's chi‐squared test.

^b^ Wilcoxon rank sum test.

^c^ Welch two sample *t*‐test.

**FIGURE 1:**
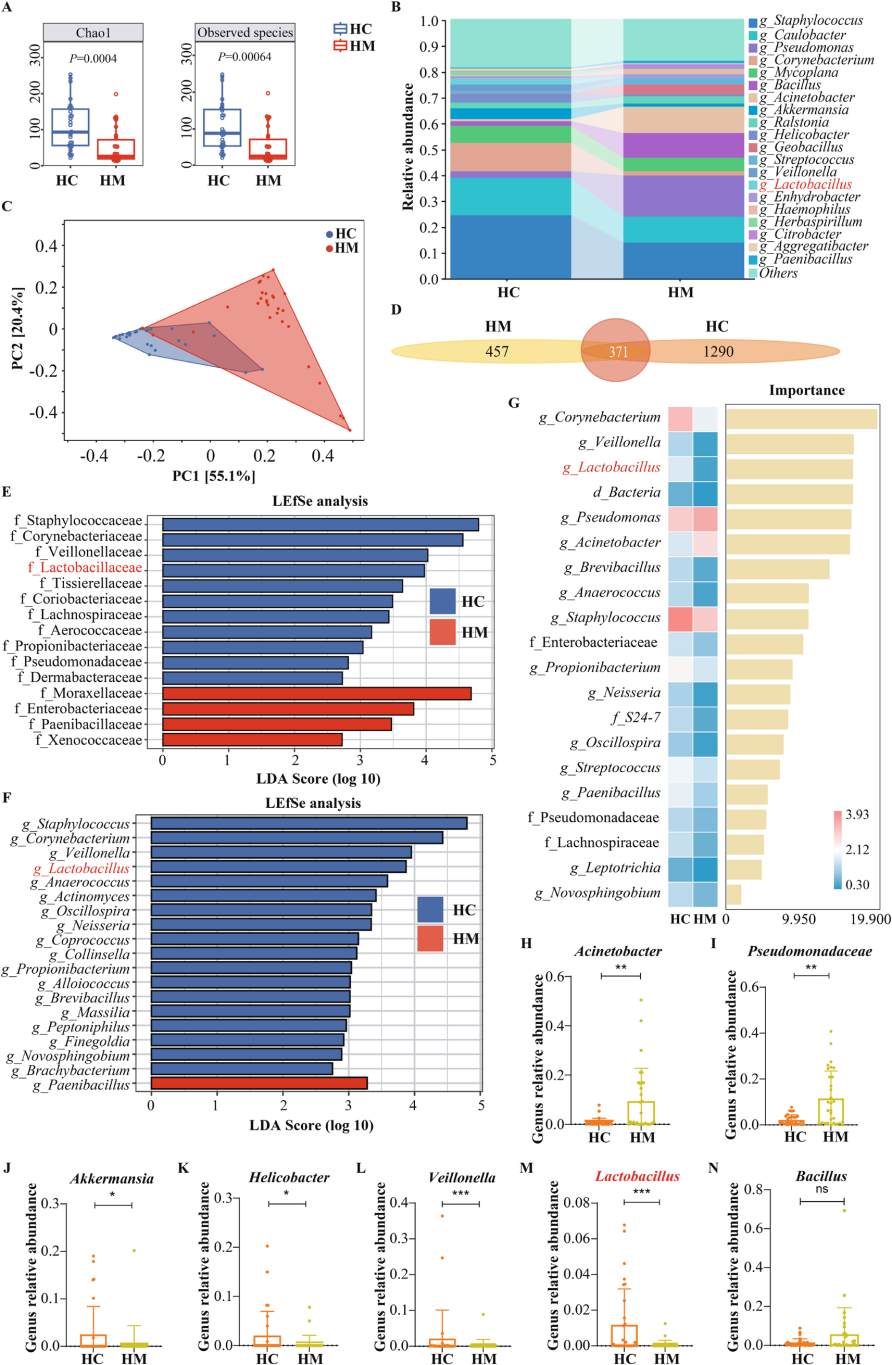
Differences in the conjunctival sac *Lactobacillus* may be a potential factor of HM. (A) Alpha diversity index. (B) Relative abundance of Top 20 bacterial genera. (C) PCA of microbial community structure. (D) Venn diagram of shared and unique microbial taxa. (E) LEfSe analysis identifies family‐level discriminative bacterial taxa. (F) LEfSe analysis identifies genus‐level discriminative bacterial taxa. (G) Random forest analysis. (H–N) Genus‐level relative abundance differences. HM, high myopia (*n* = 30). HC, healthy control (*n* = 30). Data are presented as mean ± standard deviation or median (IQR). Statistical significance was assessed by the independent samples *t*‐test, or Mann–Whitney *U* test. *p < 0.05, **p < 0.01, ***p < 0.001. ‘ns’ was no significance.

Then, high‐throughput 16S rRNA sequencing was performed to assess the conjunctival sac microbiota differences between the 2 groups. The analysis revealed a significant reduction in α‐diversity in the HM group compared to the HC group (Observed species: *p* = 0.00064; Chao1: *p* = 0.0004) (Figure 1A). Venn diagram and principal component analysis (PCA) were employed to reveal structural differences in the microbial communities between groups (Figure 1C,D), while taxonomic composition analysis was conducted to identify specific differential bacterial genera, revealing the *Lactobacillus* genus as a distinct taxon (Figure 1B). Further, Linear Discriminant Analysis Effect Size (LEfSe) was performed to identify taxa enriched at the family and genus levels in each group, which revealed significant enrichment of both Lactobacillaceae and *Lactobacillus* in the HC group (Figure 1E,F). Random Forest analysis identified *Lactobacillus* as the 3rd most important genus (Figure 1G). Furthermore, relative abundance analysis showed that *Lactobacillus* was significantly enriched in the HC group (*p* < 0.001; Figure 1H–N). Spearman correlation analysis revealed negative correlations between *Lactobacillus* abundance and AL, indicating that the depletion of *Lactobacillus* may be a characteristic of myopia‐associated dysbiosis (Figure S2).

### 3.2 *Lactobacillus mucosae* MM‐1 Was Identified as the Key Bacterium in HM Through Functional Studies

To isolate potential probiotics from the conjunctival sac of healthy individuals, bacterial colonies were screened and cultured on MRS, BHI, LB and BSM media, which led to the identification of bacterial species. Based on high‐throughput sequencing results, *L. mucosae* MM‐1 and *L. plantarum* , which belong to the genus *Lactobacillus*, were selected as candidate probiotics, and their functional efficacy was evaluated using a classical human scleral fibroblast (HSF) hypoxia model. HSFs were observed under microscopy and confirmed by vimentin immunostaining (Figure S3A,B). Subsequent experiments showed that the *L. mucosae* MM‐1 group (LM group) promoted cell migration and accelerated scratch wound closure more effectively than the *L. plantarum* group (LP group), and CCK‐8 assays indicated that *L. mucosae* MM‐1 enhanced cellular metabolic activity more potently (Figure S3C–E). Furthermore, qPCR and western blot analyses revealed that *L. mucosae* MM‐1 exerted stronger effects on COL1A1 upregulation and α‐SMA downregulation at both the gene and protein expression levels than those in the LP group (Figure S3F–J). Collectively, these results suggest that *L. mucosae* MM‐1 is a key probiotic species within the genus *Lactobacillus*.

To further evaluate the in vitro probiotic properties of *L. mucosae* MM‐1, a series of assays were conducted (Figure S4A). Microscopic examination confirmed its rod‐shaped morphology, and adhesion assays demonstrated that *L. mucosae* MM‐1 adheres to human corneal epithelial cells (HCECs) (Figure S4B,C). Growth curve analysis revealed robust growth, with the strain entering the plateau phase at 20 h and reaching a peak OD_600_ of 2.213 alongside a viable count of approximately 4.57 × 10^8^ CFU/mL (Figure S4D). Antioxidant assays demonstrated substantial radical‐scavenging and reducing activities in *L. mucosae* MM‐1 (Figure S4E), specifically: 73.84% for DPPH, 80.16% for hydroxyl radical (‐OH), 84.21% for superoxide radical (O^2−^), 21.1% for Fe^2+^ chelating ability, and a total reducing power of 0.825. The bacterium sedimented completely under standard centrifugation, yielding a supernatant with an OD_600_ < 1 (Figure S4F). Meanwhile, *L. mucosae* MM‐1 inhibited the growth of common pathogens of the conjunctival sac, with inhibition zone diameters exceeding 12 mm (Figure S5A,B). Additionally, *L. mucosae* MM‐1 exhibited resistance to some antibiotics and showed no haemolytic activity (Figure S5C–E). These traits may be natural characteristics that adapt to the ocular surface microenvironment. Together with its excellent fermentation productivity, these results suggest that *L. mucosae* MM‐1 possesses favourable probiotic properties.

### 3.3 *Lactobacillus mucosae* MM‐1 Attenuates Lens‐Induced Myopia in Mice by Promoting Scleral COL1A1 Expression

**FIGURE 2:**
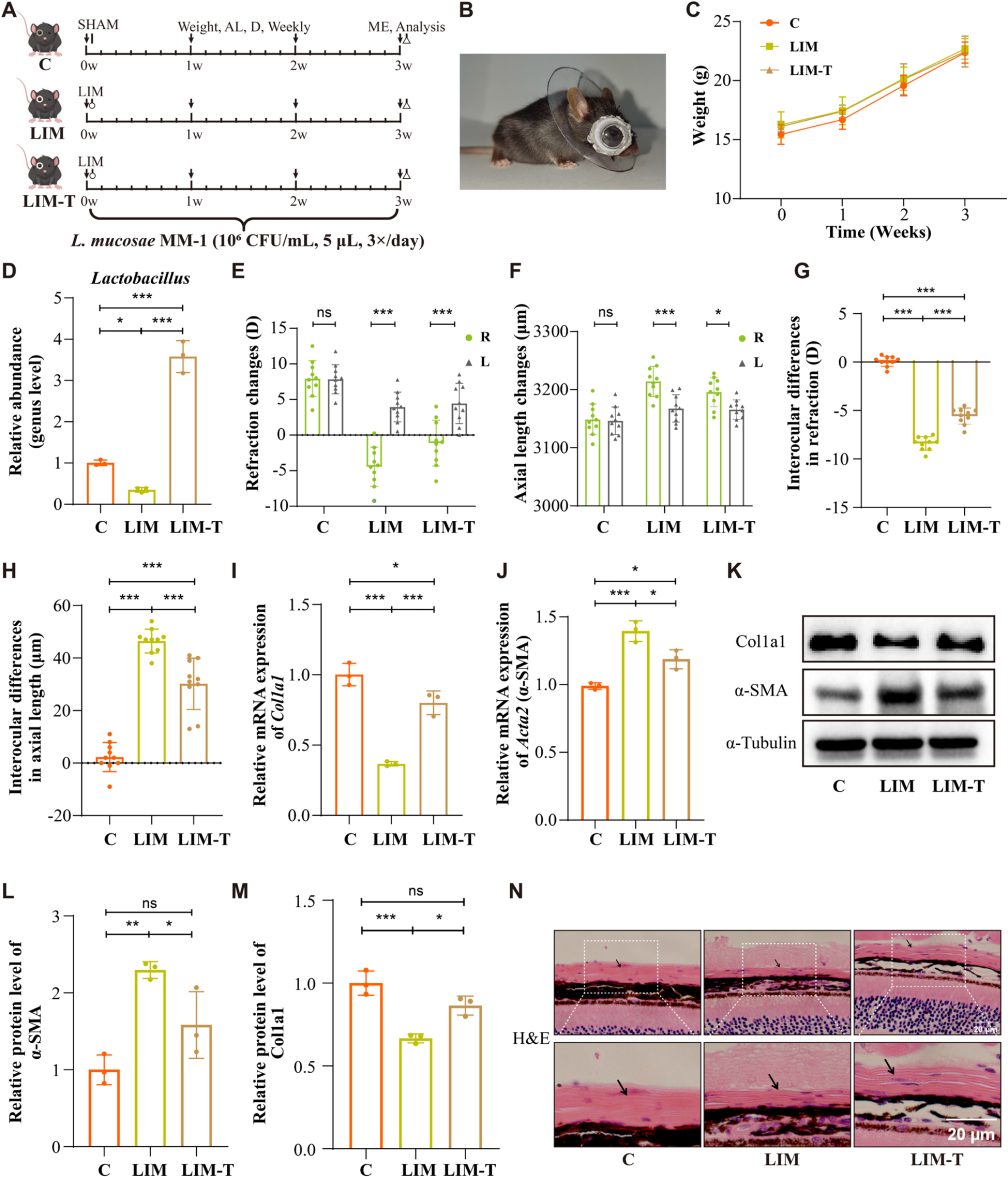
Effect of *Lactobacillus mucosae* MM‐1 on scleral remodelling and refractive progression in HM mice. (A) Research group design flowchart. See symbol legend: ↓ (time points); | (device only, no modelling); 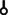 (device with HM modelling); 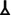 (termination/euthanasia). (B) HM modelling schematic. (C) Weekly body weight monitoring in experimental mice. (D) Genus‐level relative abundance of *Lactobacillus* (*n* = 3). (E) Post‐experimental refractive error (D) (*n* = 10). (F) Post‐experimental axial length (AL) (*n* = 10). (G) Differences in refractive error (D) (*n* = 10). (H) Differences in axial length (AL) (*n* = 10). (I) Relative mRNA expression level of scleral *Col1a1* (*n* = 3). (J) Relative mRNA expression level of scleral *Acta2* (α‐SMA) mRNA (*n* = 3). (K–M) Representative western blots and relative expression of scleral Col1a1 and α‐SMA (*n* = 3). (N) HE staining of scleral tissue (× 400; scale bar = 20 μm). C, Control group (*n* = 10), fitted with 0 D lenses and administered PBS topically to the ocular surface three times daily. LIM, lens‐induced myopia model group (*n* = 10), fitted with −30 D lenses and administered PBS topically to the ocular surface three times daily. LIM‐T, lens‐induced myopia treatment group (*n* = 10), fitted with −30 D lenses and administered probiotic solution topically to the ocular surface three times daily. Data are presented as mean ± standard deviation (SD) from independent biological replicates (*n* = 10 or 3 mice per group). Statistical significance was assessed as follows: paired data (left vs. right eyes) by two‐way ANOVA followed by Šidák's multiple comparisons test; unpaired data by one‐way ANOVA followed by Tukey's post hoc test. All comparisons were pre‐planned. *p < 0.05, **p < 0.01, ***p < 0.001. ‘ns’ indicates no significant difference (*p* ≥ 0.05).

To investigate the effect of *L. mucosae* MM‐1 on refractive error and AL progression, an HM mouse model was established. The experimental design and a schematic of the modelling apparatus are shown in Figure 2A,B. The 1.8 g lens‐wearing device was well tolerated, with no adverse effects on feeding behaviour or body weight observed (Figure 2C). The success of the *L. mucosae* MM‐1 intervention was evidenced by a 9.1‐fold increase in *Lactobacillus* abundance in the lens‐induced myopia treatment (LIM‐T) versus lens‐induced myopia model (LIM) group (Figure 2D). The LIM group developed a significant interocular refractive difference (> −6.00 D), confirming successful establishment of the HM model (Figure 2E,G). Compared to the LIM group, the LIM‐T group exhibited significant attenuation of lens‐induced myopia (Figure 2E–H). This protective effect was evidenced by a 33.6% reduction in the interocular refractive error shift (−5.58 D vs. −8.40 D; *p* < 0.001) and a 35.1% suppression of axial elongation (30.20 μm vs. 46.50 μm; *p* < 0.001) (Figure 2G,H). H&E staining further revealed that, relative to the LIM group, the scleral collagen fibre bundles in the LIM‐T group were more regularly arranged and exhibited a thickness approaching normal levels (Figure 2N). Compared to the LIM group, the LIM‐T group showed a significant increase in Col1a1 but a decrease in α‐SMA. (Figure 2I–M). Collectively, these results demonstrate that *L. mucosae* MM‐1 intervention improved scleral structure and attenuated lens‐induced myopia in mice.

### 3.4 Supernatant‐Derived ILA Is the Key Metabolite Mediating the Effects of *L. mucosae* MM‐1

To identify the key components of *L. mucosae* MM‐1 responsible for the observed anti‐myopic effects, an HSF hypoxia model was established to evaluate the impact of both bacterial supernatant and bacterial cellular components on cellular functions and ECM remodelling. Scratch assay and CCK‐8 proliferation assay were used to evaluate the migration ability and proliferative activity of scleral fibroblasts following intervention; these improvements in cellular functions serve as the cytological basis that enables ECM remodelling to occur. The results showed that compared to the bacterial cell treatment (BCT) group, the bacterial supernatant (BST) group exhibited enhanced cell migration, accompanied by a significantly higher proliferation rate in CCK‐8 assays (Figure S6A–E). Further qPCR and western blot analyses confirmed that the BST group exhibited significantly enhanced COL1A1 upregulation and α‐SMA downregulation at both the gene and protein expression levels relative to the BCT group (Figure S6F–J). Collectively, these findings indicate that the bacterial supernatant is the primary functional component mediating *L. mucosae* MM‐1‐induced cell migration and proliferation.

**FIGURE 3:**
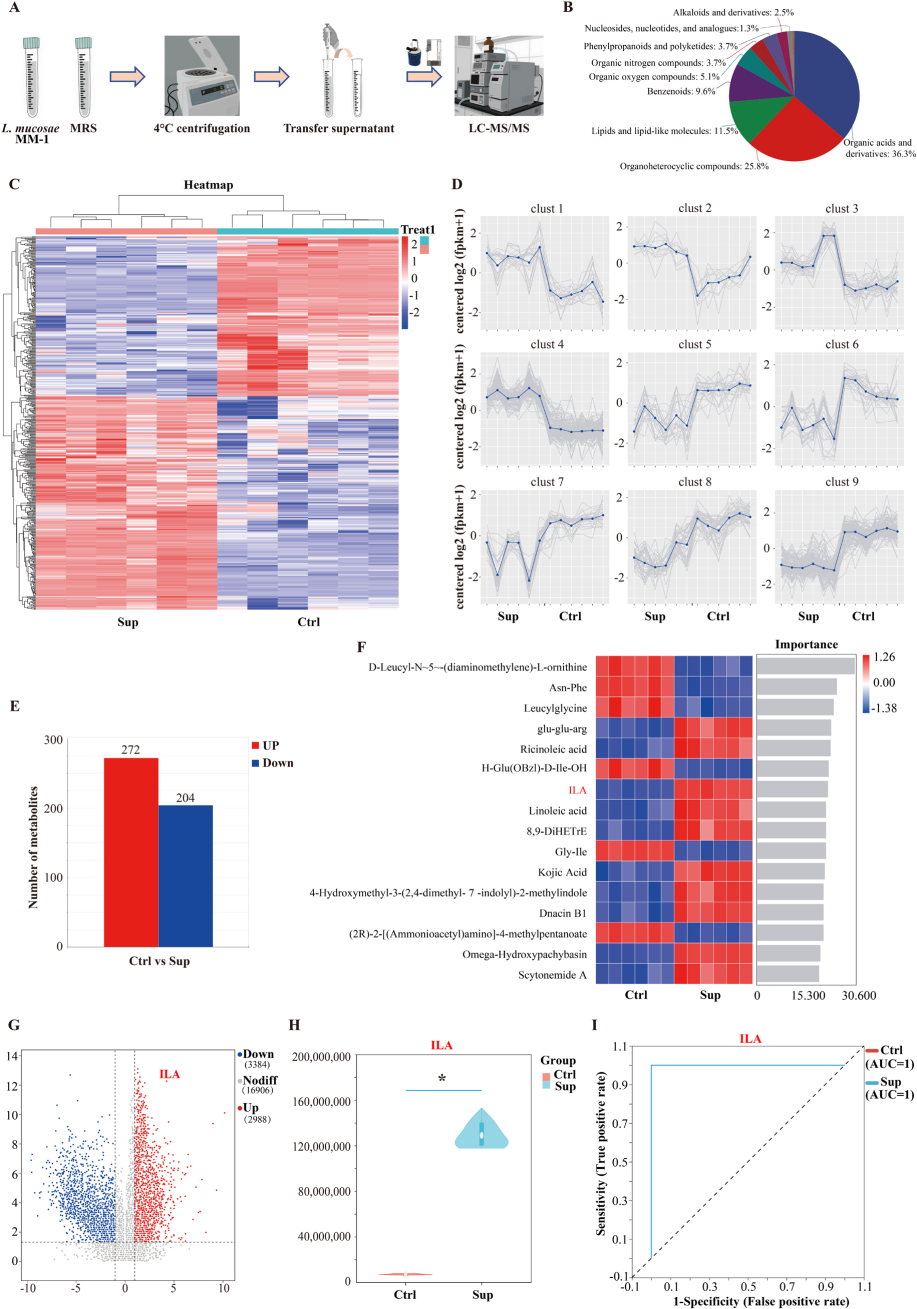
Metabolomic analysis for acquisition of probiotic metabolite composition. (A) Sample processing workflow (collection, separation and detection). (B) Metabolite identification and classification (pie chart of chemical classes). (C) Hierarchical clustering heatmap of differential metabolites. (D) Expression trend analysis of differential metabolites. (E) Screening of differential metabolites (VIP > 1 & *p* < 0.05). (F) Machine learning‐based analysis of differential substances. (G) Volcano plot of differential metabolites. (H) Differential abundance of ILA. (I) Diagnostic ROC analysis of ILA. Ctrl, MRS medium control group (*n* = 6). Sup, *Lactobacillus mucosae* MM‐1 supernatant group (*n* = 6). Data are presented as mean ± standard deviation. Statistical significance between the Ctrl and Sup groups was assessed using appropriate parametric or non‐parametric tests. *p < 0.05.

To identify the key substances in the bacterial supernatant, an untargeted metabolomic analysis was conducted with the workflow shown in Figure 3A. Data quality was confirmed by the low variance in quality control total ion chromatograms (QC‐TICs), their tight clustering in PCA and low relative standard deviations (RSDs) (Figure S7A–C), while a robust, non‐overfitting orthogonal projections to latent structures‐discriminant analysis (OPLS‐DA) model revealed clear group separation (Figure S7D,E). The identified metabolites were primarily categorised as organic acids and derivatives (36.3%) and organoheterocyclic compounds (25.8%) (Figure 3B). Compared to the MRS medium control (Ctrl) group, 272 metabolites were elevated in the *L. mucosae* MM‐1 supernatant (Sup) group (Figure 3E). Hierarchical clustering of these differential metabolites segregated them into distinct clusters based on expression patterns, with the average expression level of each cluster differing significantly between the 2 groups (Figure 3C,D). Among these, machine learning analysis highlighted ILA as a top‐ranking metabolite crucial for group discrimination (Figure 3F). The significant upregulation of ILA in the Sup group was consistently confirmed by univariate statistical analysis in the volcano plot (Figure 3G) and by a violin plot showing a significant difference in its abundance (*p* < 0.05; Figure 3H). And ILA yielded an area under the curve (AUC) of 1.0 in receiver operating characteristic (ROC) analysis, suggesting potential discriminative ability in this exploratory analysis, but this finding requires independent validation and should not be overinterpreted (Figure 3I). Further ELISA analysis showed that ILA was enriched in the bacterial supernatant, with a concentration of approximately 59.79 ng/mL (Figure S7F). 3‐IAA is a well‐characterised gut microbiota‐derived tryptophan metabolite that has been reported to suppress HM progression by promoting type I collagen synthesis. Compared with 3‐IAA, ILA showed superior efficacy in promoting fibroblast migration/proliferation, upregulating COL1A1 and downregulating α‐SMA, suggesting stronger bioactivity in modulating scleral fibroblast function (Figure S8A–H). Following *L. mucosae* MM‐1 intervention, ILA levels in the ocular surface tissues were significantly higher than those in the C group and the LIM group (Figure S9A–D). These results establish that a significant upregulation of ILA is a characteristic feature of the Sup group, identifying it as a key metabolite through our integrated analytical approach.

### 3.5 Pharmacological Evidence for AHR/TGF‐β/Smad Signalling in ILA‐Mediated COL1A1 Regulation

**FIGURE 4:**
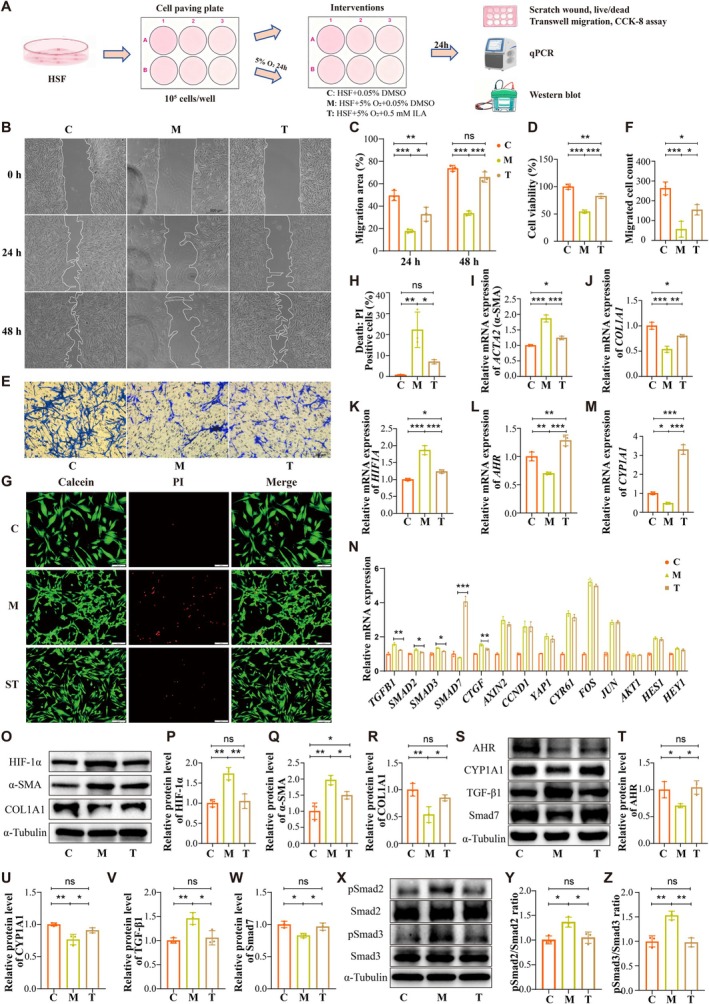
Screening for potential signalling pathways involved in ILA‐mediated regulation of COL1A1 expression. (A) Schematic diagram of experimental design. (B) Representative images of the cell scratch assay at 0, 24 and 48 h after intervention (40 × magnification; scale bars: 500 μm). (C) Quantified HSF migration area at 24 and 48 h. (D) Evaluating cell viability with CCK assay. (E, F) Transwell analysis of changes in cell migration ability (100 × magnification; scale bars: 200 μm). (G, H) Observation and evaluation of cell survival state via live/dead cell staining. (I–M) Relative mRNA expression level of *ACTA2* (α‐SMA), *COL1A1*, *HIF1A*, *AHR*, *CYP1A1* mRNA. (N) Relative mRNA expression levels of genes in related signalling pathways. (O–R) Western blot analysis of HIF‐1α, α‐SMA and COL1A1 expression. (S–W) Western blot analysis of AHR, CYP1A1, TGF‐β1 and Smad7 expression. (X–Z) Western blot analysis of pSmad2, Smad2, pSmad3 and Smad3 expression. C: Control group (*n* = 3), HSF + 0.05% DMSO; M: Model group (*n* = 3), HSF + 5% O_2_ + 0.05% DMSO; T: Treatment group (*n* = 3), HSF + 5% O_2_ + 0.5 mM ILA. Data are presented as mean ± SD from three independent biological replicates (*n* = 3 per group). Statistical significance among the three groups was assessed by one‐way ANOVA followed by Tukey's post hoc test for multiple comparisons. All comparisons were pre‐planned and unpaired. *p < 0.05, **p < 0.01, ***p < 0.001. ‘ns’ indicates no significant difference (*p* ≥ 0.05).

To investigate the effects of ILA on cellular functions, an HSF hypoxia model was established (Figure 4A). Compared with the model group (M), ILA treatment (T) significantly enhanced cell migration, as evidenced by increased wound closure in scratch assays and a greater number of migrated cells in Transwell assays (Figure 4B,C,E,F). ILA also promoted cell viability and proliferation relative to the M group, which was supported by live/dead staining and CCK‐8 assays (Figure 4D,G,H). Successful hypoxia induction was confirmed by the upregulation of HIF1A in the M group versus the control (C) group (Figure 4K,O,P). Following ILA treatment, COL1A1 expression was elevated, while the level of ACTA2 (α‐SMA) was significantly reduced compared to the M group, further indicating the inhibition of fibroblast‐to‐myofibroblast transition (FMT) (Figure 4I,J,O–R). ILA treatment also activated the AHR pathway, as evidenced by the increased expression of AHR and CYP1A1 at both the gene and protein levels (Figure 4L,M,S–U). qPCR profiling revealed a distinct expression signature in the T group relative to the M group, characterised by downregulation of *TGFB1*, *SMAD2/3* and *CTGF* accompanied by upregulation of *SMAD7*, suggesting involvement of the TGF‐β/Smad axis (Figure 4N). Western blot analysis confirmed that ILA upregulated Smad7, and downregulated TGF‐β1 and p‐Smad2/3 (Figure 4S–Z). Together, these results suggest that pharmacological engagement of AHR by ILA suppresses canonical TGF‐β/Smad signalling, accompanied by elevated COL1A1 expression and inhibition of FMT.

**FIGURE 5:**
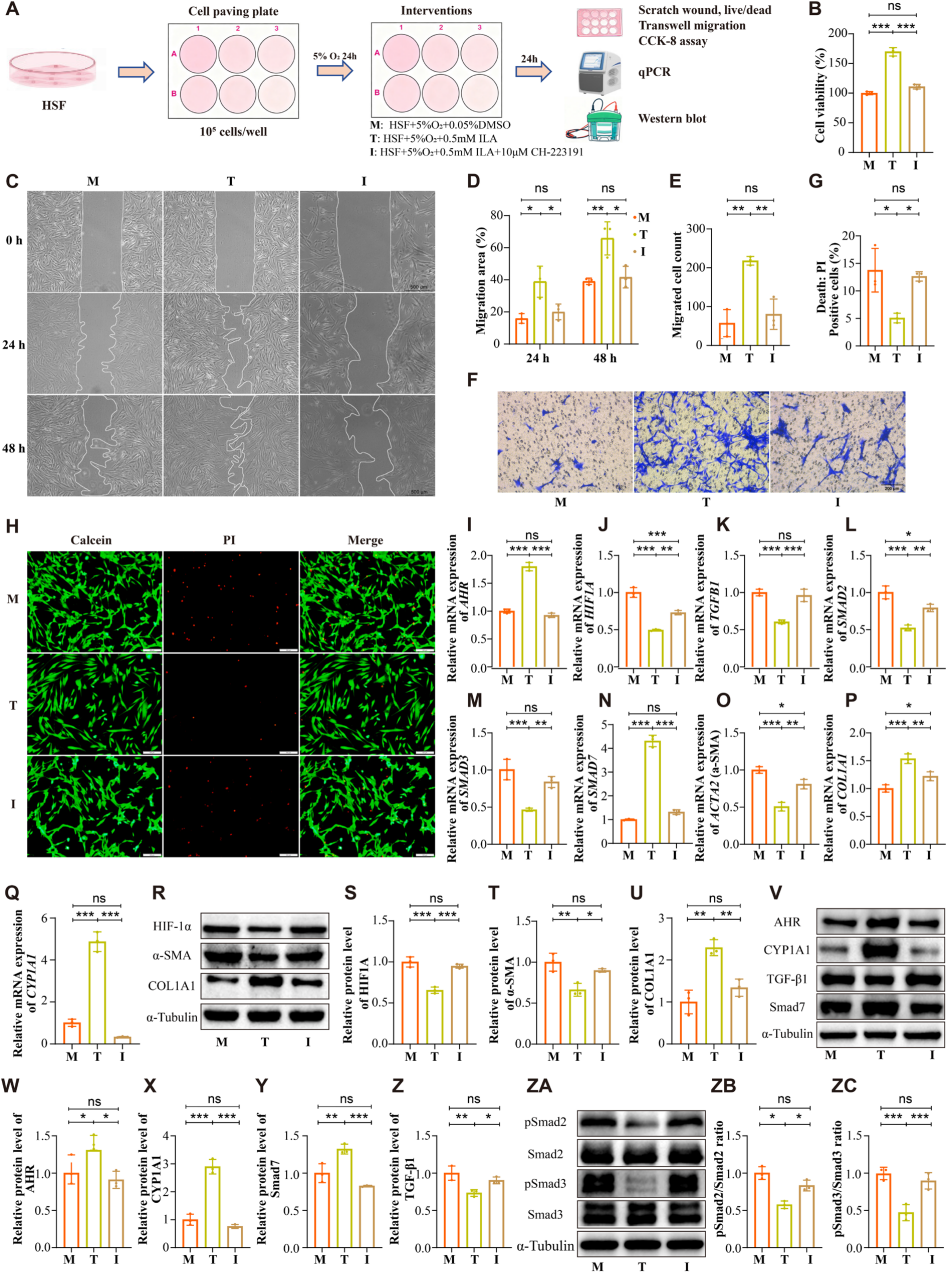
Inhibition of AHR abrogates the suppressive effect of ILA on TGF‐β/Smad signalling. (A) Schematic diagram of experimental design. (B) Evaluating cell viability with CCK assay. (C) Representative images of the cell scratch assay at 0, 24 and 48 h after intervention (40 × magnification; scale bars: 500 μm). (D) Quantified HSF migration area at 24 and 48 h. (E, F) Transwell analysis of changes in cell migration ability (100 × magnification; scale bars: 200 μm). (G, H) Observation and evaluation of cell survival state via live/dead cell staining. (I–Q) Relative mRNA expression level of *AHR*, *HIF1A*, *TGFB1, SMAD2, SMAD3, SMAD7, ACTA2* (α‐SMA), *COL1A1* and *CYP1A1* mRNA. (R–U) Western blot analysis of HIF‐1α, α‐SMA and COL1A1 expression. (V–Z) Western blot analysis of AHR, CYP1A1, TGF‐β1 and Smad7 expression. (ZA–ZC) Western blot analysis of pSmad2, Smad2, pSmad3 and Smad3 expression. M: Model group (*n* = 3), HSF + 5% O_2_ + 0.05% DMSO; T, ILA‐treated group (*n* = 3), HSF + 5% O_2_ + 0.5 mM ILA; I, AHR inhibitor group (*n* = 3), HSF + 5% O_2_ + 0.5 mM ILA + 10 μM CH‐223191. Data are presented as mean ± SD from three independent biological replicates (*n* = 3 per group). Statistical significance among the three groups was assessed by one‐way ANOVA followed by Tukey's post hoc test for multiple comparisons. All comparisons were pre‐planned and unpaired. *p < 0.05, **p < 0.01, ***p < 0.001. ‘ns’ indicates no significant difference (*p* ≥ 0.05).

To confirm whether the protective effects of ILA depend on AHR, AHR inhibitors were applied and changes in cellular functions were evaluated (Figure 5A). Compared with the M group, the T group significantly enhanced cell migration, invasion, viability and proliferation, as demonstrated by scratch wound closure, Transwell migration, live/dead staining and CCK‐8 assays (Figure 5B–H). However, these enhancements were effectively abolished by AHR inhibition (the I group), indicating AHR‐dependent mechanisms. At the molecular level, qPCR analysis revealed that, relative to the T group, the I group exhibited downregulated mRNA expression of *AHR*, *CYP1A1, SMAD7* and *COL1A1*, but upregulated levels of *HIF1A*, *TGFB1*, *SMAD2*, *SMAD3* and *ACTA2* (α‐SMA) (Figure 5I–Q). Western blotting further confirmed that AHR inhibition reduced protein levels of COL1A1, AHR, CYP1A1 and Smad7, while increasing HIF‐1α, α‐SMA, TGF‐β1 and p‐Smad2/3 (Figure 5R–ZC).

Therefore, pharmacological ILA supplementation supports the involvement of AHR in TGF‐β/Smad pathway inhibition, along with altered COL1A1 and α‐SMA expression.

### 3.6 AHR Inhibition Abrogates the Protective Effect of *L. mucosae* MM‐1 or ILA on HM and TGF‐β/Smad Signalling

**FIGURE 6:**
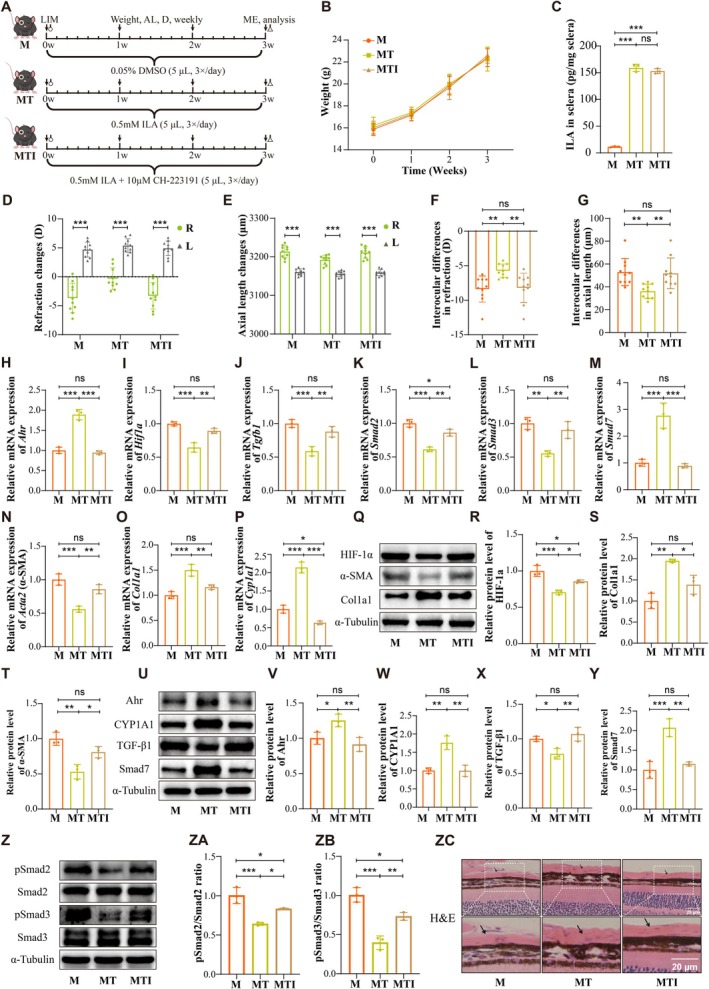
AHR inhibition abrogates the protective effect of ILA against lens‐induced myopia in mice by disrupting ILA‐mediated TGF‐β/Smad suppression. (A) Research group design flowchart. See symbol legend: ↓ (time points); | (device only, no modelling); 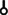 (device with HM modelling); 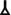 (termination/euthanasia). (B) Weekly body weight monitoring in experimental mice. (C) Quantification of ILA levels in the sclera of mice by ELISA (*n* = 3). (D) Post‐experimental refractive error (D) (*n* = 10). (E) Post‐experimental axial length (AL) (*n* = 10). (F) Differences in refractive error (D) (*n* = 10). (G) Differences in axial length (AL) (*n* = 10). (H–P) Relative mRNA expression level of scleral *Ahr*, *Hif1α*, *Tgfb1*, *Smad2*, *Smad3*, *Smad7*, *Acta2* (α‐SMA), *Col1a1* and *Cyp1a1* (*n* = 3). (Q–T) Western blot analysis of HIF‐1α, α‐SMA and Col1a1 expression (*n* = 3). (U–Y) Western blot analysis of Ahr, CYP1A1, TGF‐β1 and Smad7 expression (*n* = 3). (Z–ZB) Western blot analysis of pSmad2, Smad2, pSmad3 and Smad3 expression (*n* = 3). (ZC) HE staining of scleral tissue (×400; scale bar = 20 μm). M: Model group (*n* = 10), fitted with −30 D lenses and administered 5 μL of 0.05% DMSO topically to the ocular surface three times daily. MT: Metabolite treatment group (*n* = 10), fitted with −30 D lenses and administered 5 μL of 0.5 mM ILA in 0.05% DMSO topically to the ocular surface three times daily. MTI: Metabolite and inhibitor treatment group (*n* = 10), fitted with −30 D lenses and administered 5 μL of the mixture (0.5 mM ILA and 10 μM CH‐223191 in 0.05% DMSO) topically to the ocular surface three times daily. Data are presented as mean ± standard deviation (SD) from independent biological replicates (*n* = 10 or 3 mice per group). Statistical significance was assessed as follows: Paired data (left vs. right eyes) by two‐way ANOVA followed by Šidák's multiple comparisons test; unpaired data by one‐way ANOVA followed by Tukey's post hoc test. All comparisons were pre‐planned. *p < 0.05, **p < 0.01, ***p < 0.001. ‘ns’ indicates no significant difference (*p* ≥ 0.05).

To evaluate the in vivo pharmacological effect of exogenous ILA supplementation on myopia, an HM mouse model was established (Figure 6A). No significant difference in body weight was observed among the groups (Figure 6B). Scleral ILA concentration was significantly elevated in the metabolite treatment (MT) and metabolite and inhibitor treatment (MTI) groups (approximately 13.95‐ and 13.42‐fold, respectively) relative to the model (M) group (*p* < 0.001; Figure 6C), confirming successful local delivery. After ILA treatment, the scleral ILA concentration reached approximately 153.0 pg/mg (Figure 6C). Compared with the M group, the MT group exhibited a significantly slower progression of myopia, as indicated by a less myopic refractive error and slowed AL elongation (Figure 6D–G). Notably, co‐administration of an AHR inhibitor (MTI group) largely abolished these protective effects. Specifically, compared to the MT group, the MTI group exhibited a 43.1% increase in interocular refractive error (−8.20 D vs. −5.73 D; *p* < 0.01) and a 43.2% greater AL disparity (51.85 μm vs. 36.20 μm; *p* < 0.01) (Figure 6F,G). At the molecular level, qPCR analysis showed that ILA treatment significantly upregulated *Ahr*, *CYP1A1, Smad7* and *Col1a1*, while downregulating *Hif1α*, *Tgfb1*, *Smad2*, *Smad3* and *Acta2* (α‐SMA) compared to the M group (Figure 6H–P). These changes were reversed in the MTI group, indicating AHR dependence. Western blotting confirmed that AHR inhibition reduced protein levels of Ahr, CYP1A1, Smad7 and Col1a1, while elevating HIF‐1α, TGF‐β1, pSmad2/3 and α‐SMA relative to the MT group (Figure 6Q–ZB). HE staining revealed that the improved scleral thickness and collagen organisation observed in the MT group were reversed in the MTI group, which exhibited a thinned and disorganised sclera similar to the M group (Figure 6ZC). In summary, these in vivo results provide pharmacological evidence supporting the involvement of AHR in the ILA‐mediated alleviation of myopic scleral remodelling, an effect that correlates with inhibition of the TGF‐β/Smad pathway, upregulation of COL1A1 and downregulation of ACTA2 (α‐SMA) expression.

**FIGURE 7:**
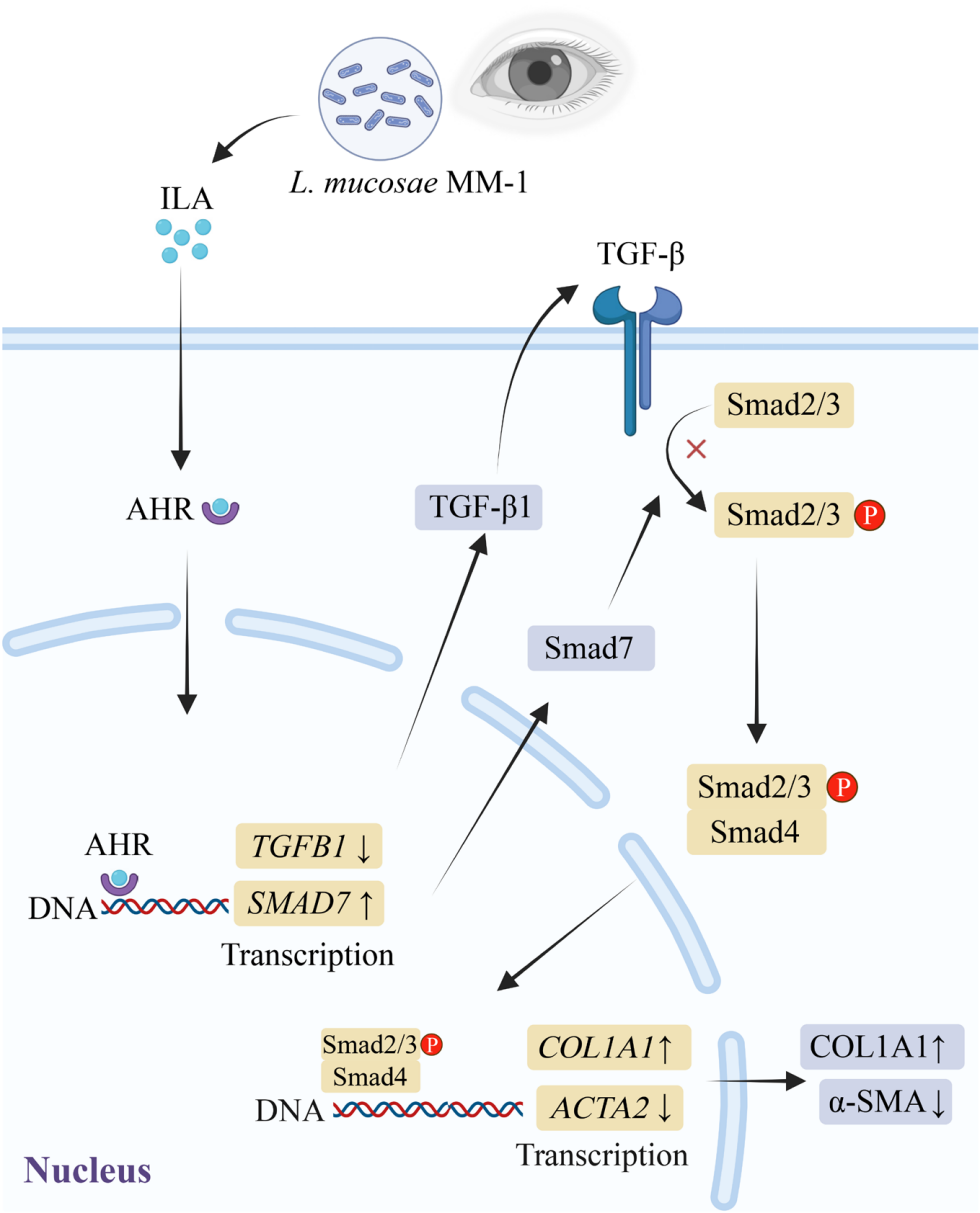
Schematic diagram illustrating the potential involvement of *Lactobacillus mucosae* MM‐1‐derived ILA in attenuating lens‐induced myopia in mice with associated modulation of the AHR/TGF‐β/Smad axis. This figure was adapted using elements from BioRender (https://biorender.com).

To investigate the molecular mechanism underlying the protective effect of *L. mucosae* MM‐1 against lens‐induced myopia in mice, we co‐administered *L. mucosae* MM‐1 with the AHR inhibitor CH223191. As shown in Figure S10A, CH223191 effectively abolished the in vivo beneficial effects of *L. mucosae* MM‐1. No significant differences in body weight gain were observed among all groups during the intervention period (Figure S10B). Measurements of refractive error and axial length revealed that the LTI group exhibited significantly increased myopic shifts and axial elongation compared with the LT group, reaching levels comparable to those of the M group (Figure S10C–F). At the molecular level, relative to the LT group, the LTI group showed markedly decreased expression of AHR, its downstream target CYP1A1, the inhibitory Smad7 and Col1a1, accompanied by significantly elevated levels of HIF‐1α, pSmad2/3 and α‐SMA (Figure S10G–ZA). Histologically, HE staining demonstrated that the LTI group displayed aggravated scleral remodelling with reduced scleral thickness, resembling the M group phenotype (Figure S10ZB). Collectively, these results provide pharmacological evidence supporting the involvement of the AHR signalling pathway in the protective effects of *L. mucosae* MM‐1. The mechanism is illustrated in Figure 7.

## 4 Discussion

Currently, HM is a prevalent refractive error globally, particularly among adolescents, making it imperative to develop effective prevention and control strategies. With progressing research on the conjunctival sac microbiota, accumulating evidence suggests its potential role in the onset, progression and treatment of HM. However, the specific microorganisms involved and their precise mechanisms of action remain poorly understood. Therefore, investigating the role of the conjunctival sac microbiota in HM is essential for elucidating its pathogenesis and developing novel interventions.

To answer the above question, this study first compared the conjunctival sac microbiota between healthy individuals and HM patients. The results showed that compared with healthy individuals, the abundance of *Lactobacillus* and the α‐diversity in HM patients decreased, while the abundance of *Acinetobacter* increased (Figure 1). Furthermore, correlation analyses revealed a negative association between *Lactobacillus* abundance and key myopia indicators, suggesting *Lactobacillus* might be a core functional bacterium (Figure S2). Subsequently, two *Lactobacillus* strains were isolated from healthy conjunctival sacs via culturomics and evaluated in vitro cell‐based assays, leading to the identification of strain *L. mucosae* MM‐1 as the core functional *Lactobacillus*. *Lactobacillus* is a commensal probiotic genus that inhabits the ocular surface, gut and vagina, and plays a crucial role in maintaining health. Studies indicate that HM and dry eye disease patients exhibit dysbiosis of the conjunctival sac microbiota and a significant reduction in *Lactobacillus* abundance (Gupta et al. 2023; Xiao et al. 2023; Zhao et al. 2025), suggesting that *Lactobacillus* might play a protective role in ocular surface homeostasis and myopia progression.

Building on the integration of culturomics and human metagenomic sequencing, we identified two candidate strains and ultimately selected *L. mucosae* MM‐1 for further study. Subsequent in vitro assays confirmed its favourable probiotic profile, demonstrating notable antioxidant capacity, inhibition of pathogenic bacterial growth, and a negative haemolytic response (Figures S4 and S5). To evaluate its therapeutic potential in vivo, we employed an HM mouse model of lens‐induced myopia (Li et al. 2024; Tkatchenko et al. 2010). In this model, myopia is induced by a −30 D negative lens that displaces the focal plane behind the retina, triggering compensatory axial elongation that mimics human myopia development (Wallman and Winawer 2004). Myopia progression is accompanied by decreased scleral COL1A1 expression, enhanced FMT, pathological ECM remodelling, scleral thinning and axial elongation (McBrien 2003). The scleral remodelling marker α‐SMA (a myofibroblast‐specific protein) can indirectly reflect the ECM remodelling and is widely used in mechanistic studies of myopia‐related scleral remodelling (Zhao et al. 2025). Our results indicate that administration of *L. mucosae* MM‐1 improved myopia‐related parameters, alleviated scleral remodelling, elevated COL1A1 expression and suppressed the myopic marker α‐SMA (Figure 2). These findings are consistent with previous reports on probiotic eye drops for myopia intervention, including a *Lactobacillus*‐based eye drop that effectively delayed the myopic phenotype, suppressed α‐SMA expression and modulated scleral COL1A1 expression (Zhao et al. 2025). Mechanistically, *L. mucosae* was reported to modulate IL‐1β, TNF‐α and TGF‐β levels, while inhibiting AKT, NF‐κB and STAT3 pathways (Hao et al. 2023; Li et al. 2023; Sun et al. 2024; Zhang et al. 2022). Through these mechanisms, it may alleviate intestinal and neuroinflammation, exert antiviral effects, and potentially modulate myopia progression (Ma et al. 2023; Wang et al. 2022). Therefore, *L. mucosae* MM‐1 is a beneficial probiotic that effectively attenuated lens‐induced myopia in mice.

To identify the core component of *L. mucosae* MM‐1, we separated the supernatant and bacterial pellet via centrifugation and evaluated them in cell‐based assays, revealing the supernatant to be the primary functional component (Figure S6). Since metabolites in the supernatant are central mediators of microbiota function (Boyajian et al. 2024; Shan et al. 2023; Zheng‐Qiang et al. 2025), we performed untargeted metabolomic analysis and identified ILA as the key metabolite (Figure 3). ILA is a tryptophan metabolite that exhibits versatile biological activities. It can modulate the AHR pathway, exert anti‐inflammatory and immunoregulatory effects (Groeger et al. 2025; Liu et al. 2025), alleviate neuroinflammation and depression‐like behaviours (Shu et al. 2026; Wang et al. 2025) and enhance intestinal barrier function (Liu et al. 2025). Mechanistically, ILA downregulates the glycolysis, NF‐κB, NLRP3 and HIF signalling pathways (Yu et al. 2023; Zhang et al. 2025), all of which play important roles in the progression of myopia (Lin et al. 2025, 2024). Collectively, these results demonstrate that ILA, enriched in the *L. mucosae* MM‐1 supernatant, is likely the major mediator of its functional effects.

Furthermore, the hypoxic HSF model was employed to investigate the mechanism of ILA's effect on myopia progression (Figure 4). This model is widely employed in myopia research due to its ability to mimic the scleral conditions associated with myopia (Lin et al. 2024; Pan et al. 2021; Xue et al. 2024; Zhao et al. 2020). The experimental results demonstrated that, compared with the hypoxic model group, ILA treatment significantly enhanced cell viability, suppressed α‐SMA and HIF‐1α expression and promoted COL1A1 and AHR expression, confirming its protective role against myopia‐related pathological remodelling at the cellular level. This pattern is consistent with the established role of AHR in suppressing the HIF‐1α pathway (Bahman et al. 2024; Salminen 2022). In addition, to identify the target genes of ILA, RT‐qPCR was used to analyse the expression of multiple genes involved in ECM remodelling and myopia‐associated signalling pathways. Compared with the hypoxic model group, ILA intervention significantly downregulated *TGFB1*, *SMAD2*, *SMAD3* and *CTGF*, while upregulating *SMAD7*. These findings are consistent with numerous studies that underscore the crucial role of the TGF‐β/Smad pathway in myopia progression. Although this pathway primarily promotes fibrosis in heart and liver (Yang et al. 2022), it exhibits a distinct mode of action in the myopic sclera (Xie et al. 2020). In scleral fibroblasts, TGF‐β activates downstream Smad proteins upon receptor binding, which inhibit COL1A1 synthesis, promote ECM degradation and drive pathological FMT (Xie et al. 2020; Zhao et al. 2025). Consistent with this, inhibition of TGF‐β pathway activity in a lens‐induced myopia guinea pig model attenuated scleral remodelling and restrained abnormal increases in refractive error and AL (Zhu et al. 2025). In this study, ILA may act through two parallel mechanisms: upregulating Smad7 to reinforce TGF‐β/Smad negative feedback and alleviating hypoxia, which in turn downregulates HIF‐1α to relieve COL1A1 transcriptional suppression, thus restoring ECM homeostasis. Based on this evidence, we hypothesised that ILA may activate AHR to inhibit the TGF‐β/Smad signalling pathway, thereby promoting COL1A1 expression and ultimately reversing ECM remodelling.

To test this hypothesis, we analysed key molecules in the TGF‐β/Smad pathway, which showed that ILA treatment significantly activated AHR, modulated key proteins (TGF‐β1, Smad7, pSmad2, pSmad3, α‐SMA), and increased COL1A1 levels compared to the hypoxic model group. These findings align with prior reports that certain tryptophan metabolites can activate AHR and inhibit the TGF‐β/Smad pathway (Gramatzki et al. 2009; Liu et al. 2020; Luo et al. 2024). Moreover, IAA has been reported to promote scleral COL1A1 expression and delay HM progression (Li et al. 2024). In this study, ILA was identified as a differential metabolite through metabolomics, whereas 3‐IAA was not a significant differential metabolite. Compared with 3‐IAA, ILA showed superior efficacy in promoting fibroblast migration/proliferation, upregulating COL1A1 and downregulating α‐SMA, suggesting stronger bioactivity in modulating scleral fibroblast function (Figure S8). Collectively, these results suggest that ILA improves scleral remodelling through AHR engagement, which correlates with modulation of the TGF‐β/Smad signalling pathway.

Finally, to validate the role of the AHR/TGF‐β/Smad signalling pathway in myopia control, we applied the AHR inhibitor CH223191 in both cellular and animal experiments (Figures 5 and 6). CH223191 is a potent and selective AHR inhibitor that has been widely used to investigate AHR‐related signalling pathways (Zhang et al. 2023). In cellular experiments, AHR inhibition not only blocked ILA‐promoted cell migration and survival but also reversed its core regulatory effects at the molecular level. Specifically, it prevented the upregulation of AHR, CYP1A1, Smad7 and COL1A1, while restoring high expression levels of HIF‐1α, TGF‐β1, p‐SMAD2/3 and ACTA2 (α‐SMA). In animal studies, CH223191 abolished the attenuating effect of *L. mucosae* MM‐1 or ILA on lens‐induced myopia in mice, and this was accompanied by reduced TGF‐β/Smad signalling. These findings are consistent with previous reports that AHR inhibitors alleviate AHR‐mediated negative regulation of TGF‐β signalling (Zhang et al. 2023), further supporting the conclusion that *L. mucosae* MM‐1 or ILA suppresses the TGF‐β/Smad pathway and its associated scleral remodelling in an AHR‐dependent manner.

### 4.1 Limitations of the Study

Several limitations of this study should be acknowledged. First, although potential confounding factors were controlled through the inclusion and exclusion criteria in this clinical observational study, the findings remain insufficient to establish a causal relationship between HM and alterations in conjunctival sac microbiota composition. The observed microbiome differences may also reflect confounding by behavioural factors associated with myopia. In an animal model, intervention with *L. mucosae* MM‐1 effectively attenuated lens‐induced myopia in mice, and further studies are warranted to extend these findings. Second, in this study, ILA was quantified only by ELISA; the absolute concentrations and metabolic profiles of ILA in tissues, bacterial culture supernatant and tear fluid remain to be validated by targeted LC–MS/MS‐based metabolomics in future studies. Third, the present pharmacological evidence suggests that AHR may be involved in regulating the TGF‐β/Smad axis. However, given the absence of inhibitor‐only controls and genetic loss‐of‐function validation, these findings should be viewed with caution, and further direct genetic intervention experiments are required for verification in future research. Finally, all animals in this experimental design wore the modelling apparatus (0 D lenses for the control group and −30 D lenses for the model group). The absolute effect of the device itself on the ocular surface microenvironment was not quantitatively assessed, but this does not affect the scientific validity of the between‐group comparisons in this model.

## 5 Conclusion

In this study, high‐throughput sequencing and culturomics were used to reveal the compositional characteristics of the conjunctival sac microbiota in individuals with HM and healthy subjects. At the same time, a strain of *L. mucosae* MM‐1 with favourable probiotic properties was isolated through probiotic screening. Furthermore, animal experiments verified that *L. mucosae* MM‐1 significantly attenuates lens‐induced myopia in mice, improves both COL1A1 expression and scleral integrity, and elevates the abundance of *Lactobacillus* in the conjunctival sac. At the same time, ILA was identified as the core molecule mediating this effect. In addition, combined with animal and HSF hypoxic models along with inhibitor interventions, ILA was shown to engage AHR, correlating with inhibition of the TGF‐β/Smad signalling pathway and attenuation of lens‐induced myopia in mice. Moreover, this study is the first to propose a ‘conjunctival sac microecology‐metabolite‐scleral remodelling’ regulatory axis, highlighting the potential role of conjunctival sac microbiota in maintaining ocular surface health and regulating lens‐induced myopia in mice, providing important theoretical foundation for the developing microbiome‐based interventions for myopia control. Future studies should attempt to discover other novel strains in the conjunctival sac of healthy individuals that may also contribute to delaying myopia and maintaining ocular health.

## Author Contributions

**Qiuyu Wu:** methodology, writing – review and editing. **Luchuan Liu:** methodology, writing – review and editing. **Xilong Zhang:** methodology, writing – review and editing. **Leiping Ding:** methodology, writing – review and editing. **Jing Wei:** methodology, writing – review and editing. **Yanqing Li:** conceptualization, methodology, data curation, formal analysis, writing – original draft, writing – review and editing, visualization. **Wen Yao:** methodology, writing – review and editing. **Di Wu:** methodology, writing – review and editing. **Yifeng Yu:** conceptualization, writing – review and editing, funding acquisition. **Chiwen Cheng:** conceptualization, writing – review and editing. **Jiale Li:** methodology, writing – review and editing. **Yangyang Peng:** methodology, writing – review and editing. **Yijie Pi:** methodology, writing – review and editing. **Kang Yu:** methodology, writing – review and editing. **Jingjing Xu:** conceptualization, writing – review and editing. **Tingtao Chen:** conceptualization, writing – review and editing.

## Funding

This work was supported by the Graduate Innovation Special Fund of Jiangxi Province, China (grant YC2024‐B072).

## Ethics Statement

The clinical component of this study received approval from the Biomedical Research Ethics Committee of the Second Affiliated Hospital of Nanchang University (Approval No.: O‐Yiyan Lun Shen [2025] No. (20)). The animal experiments were approved by the Laboratory Animal Welfare Ethics Committee of Nanchang University (Approval No.: NCULAE‐20221031187).

## Conflicts of Interest

The authors declare no conflicts of interest.

## Supporting information


**Figure S1:** Study grouping flow chart.
**Figure S2:** Spearman's correlation heatmap of ocular surface microbiota and myopia‐related parameters.
**Figure S3:** The effect of ocular surface‐derived potential probiotics on HSF.
**Figure S4:** Acquisition and phenotypic features of *Lactobacillus mucosae* MM‐1.
**Figure S5:** Virulence and antibiotic resistance of *Lactobacillus mucosae* MM‐1.
**Figure S6:** Effect of bacterial secretome and cellular components on cell migration and proliferation.
**Figure S7:** Metabolomic quality control, model evaluation and ELISA‐based ILA quantitation.
**Figure S8:** ILA exhibits superior modulatory effects over 3‐IAA on scleral fibroblast functional phenotypes.
**Figure S9:** ILA concentrations in mouse ocular surface tissues after *Lactobacillus mucosae* MM‐1 intervention.
**Figure S10:** AHR inhibition abrogates the protective effect of *Lactobacillus mucosae* MM‐1 on HM progression in mice via disruption of TGF‐β/Smad suppression.
**Table S1:** PCR primer sequences.
**Table S2:** Antibodies used in this study.

## Data Availability

The raw 16S rRNA gene sequencing data from this clinical study have been deposited in the NCBI Sequence Read Archive under BioProject accession number PRJNA1399909 (https://dataview.ncbi.nlm.nih.gov/object/PRJNA1399909). The metabolomics data generated in this study have been deposited in the MetaboLights repository under study accession number MTBLS15008 (https://www.ebi.ac.uk/metabolights/MTBLS15008).
