# Supplementary material for: Conjunctival Sac‐Derived Lactobacillus mucosae MM‐1 Metabolite Indole‐3‐Lactic Acid Attenuates Lens‐Induced Myopia in Mice via AHR Activation and TGF‐β/Smad Inhibition
Source: Microb Biotechnol. 2026 Aug 7;19(8):e70426. doi: 10.1111/1751-7915.70426 (PMC13449032; doi:10.1111/1751-7915.70426)
Supplement: Supplementary file 1 [file MBT2-19-e70426-s001.docx]

**Supplementary Information**

**Figure S1** **Study grouping flow chart**

**Figure S2 Spearman's correlation heatmap of ocular surface microbiota and myopia-related parameters**

**Figure S3 The effect of ocular surface-derived potential probiotics on HSF. A.** Photomicrograph of cells (100×), scale bar 200 μm. **B.** Immunofluorescent identification of Vimentin protein in HSF (400×), scale bar 200 μm. **C.** Representative images of cell scratch assay at 0 h, 24 h, and 48 h post-treatment with LP and LM (40×), scale bar 500 μm. **D.** Quantitative analysis of HSF migration area. **E.** Assessment of cell viability following LP and LM intervention. **F-G.** Relative mRNA expression level of *ACTA2* (α-SMA) and *COL1A1*. **H-J.** Western blot analysis of α-SMA, and COL1A1 expression. **M**, model group (n=3); **LM**, *L. mucosae* MM-1 treatment after cell modeling; **LP**, *L. plantarum* treatment after cell modeling*.* Data are presented as mean ± SD from three independent biological replicates (n = 3 per group). Statistical significance among the three groups was assessed by one‑way ANOVA followed by Tukey's post hoc test for multiple comparisons. All comparisons were pre‑planned and unpaired. **P* < 0.05, ***P* < 0.01, ****P* < 0.001. "ns" indicates no significant difference (*P* ≥ 0.05).

**Figure S4 Acquisition and phenotypic features of *Lactobacillus mucosae* MM-1*.* A.** Procedure for bacterial source, identification and probiotic properties evaluation. **B.** Bacterial staining image (1000×), scale bar 20 μm. **C.** Adhesion assay of bacteria to corneal epithelial cells (1000×), scale bar 20 μm. (1) PBS control; (2) Bacterial suspension. **D.** *L. mucosae* MM-1 growth curve: viable count (log CFU/mL) and absorbance (OD_600_). **E.** Antioxidant capacity assay. **F.** Centrifugation performance. Data are presented as mean ± standard deviation.

**Figure S5 Virulence and antibiotic resistance of *Lactobacillus mucosae* MM-1*.* A.** *L. mucosae* MM-1 antibacterial properties evaluation. (1) *S. epidermidis, Staphylococcus epidermidis*; (2) *E. coli, Escherichia coli*; (3) *S. aureus*; (4) *P. mirabilis, Proteus mirabilis*; (5) *P. aeruginosa, Pseudomonas aeruginosa*. **B.** Inhibition zone diameters of *L. mucosae* MM-1 against pathogenic bacteria. **C.** Hemolysis assay. (1) *L. mucosae* MM-1; (2) *S. aureus*. **D.** Drug resistance assay. TET, Tetracycline; SXT, Trimethoprim/Sulfamethoxazole; PEN, Penicillin; MY, Lincomycin; GEN, Gentamicin; ERY/E, Erythromycin; CTR, Ceftriaxone; CIP, Ciprofloxacin; C, Chloramphenicol; AMP, Ampicillin. **E.** Suppression diameter of *L. mucosae* MM-1 against various antibiotics. Data are presented as mean ± standard deviation.

**Figure S6 Effect of bacterial secretome and cellular components on cell migration and proliferation. A-B.** Cell viability of HSF treated with increasing concentrations of *L. mucosae* MM-1 supernatant or UV-inactivated bacterial cells. **C.** Quantified HSF migration area at 24 h and 48 h. **D.** Representative images of the cell scratch assay at 0 h, 24 h, and 48 h after treatment with BST and BCT (40× magnification; scale bars: 500 μm). **E.** Evaluating cell viability with CCK assay. **F-G.** Relative mRNA expression level of *ACTA2* (α-SMA) and *COL1A1*. **H-J.** Western blot analysis of α-SMA, and COL1A1 expression. M: Model group (n=3), HSF+5%O_2_+MRS medium; BST: bacterial supernatant group (n=3), HSF+5%O_2_+bacterial supernatant; BCT: bacterial cell treatment group (n=3), HSF+5%O_2_+UV-inactivated bacterial cells. Data are presented as mean ± SD from three independent biological replicates (n = 3 per group). Statistical significance among the three groups was assessed by one‑way ANOVA followed by Tukey's post hoc test for multiple comparisons. All comparisons were pre‑planned and unpaired. **P* < 0.05, ***P* < 0.01, ****P* < 0.001. "ns" indicates no significant difference (*P* ≥ 0.05).

**Figure S7 Metabolomic quality control, model evaluation, and ELISA-based ILA quantitation.**

**A.** Overlay of QC sample mass spectrometry peaks (quality control). **B.** Principal component analysis (PCA) of all samples. **C.** QC relative standard deviation. **D.** OPLS-DA score plot showing the separation between groups. **E.** Permutation test plot for validating the OPLS-DA model. **F**. ILA concentration in the bacterial supernatant. **Ctrl**: MRS medium control group (n=6). **Sup**: *L. mucosae* MM-1 supernatant (n=6). Data are presented as mean±standard deviation or median (IQR). Statistical significance was assessed by the independent samples t-test, or Mann-Whitney U test. All comparisons were pre- planned and unpaired. **P* < 0.05, ***P* < 0.01, ****P* < 0.001. "ns" indicates no significant difference (*P* ≥ 0.05).

**Figure S8 ILA exhibits superior modulatory effects over 3-IAA on scleral fibroblast functional phenotypes. A.** Representative images of the cell scratch assay at 0 h, 24 h, and 48 h after treatment with ILA and 3-IAA (40× magnification; scale bars: 500 μm). **B.** Quantified HSF migration area at 24 h and 48 h. **C.** Evaluating cell viability with CCK assay. **D-E.** Relative mRNA expression level of *ACTA2* (α-SMA) and *COL1A1*. **F-H.** Western blot analysis of α-SMA, and COL1A1 expression. M: Model group (n=3), HSF+5%O_2_+0.05% DMSO; ILA: ILA treatment group (n=3), HSF+5%O_2_+0.5mM ILA; 3-IAA: 3-IAA treatment group (n=3), HSF+5%O_2_+0.5mM 3-IAA. Data are presented as mean ± SD from three independent biological replicates (n = 3 per group). Statistical significance among the three groups was assessed by one‑way ANOVA followed by Tukey's post hoc test for multiple comparisons. All comparisons were pre‑planned and unpaired. **P* < 0.05, ***P* < 0.01, ****P* < 0.001. "ns" indicates no significant difference (*P* ≥ 0.05).

**Figure S9 ILA concentrations in mouse ocular surface tissues after *Lactobacillus mucosae* MM-1 intervention*.* A.** ILA concentration in the cornea. **B.** ILA concentration in the conjunctiva. **C.** ILA concentration in the sclera. **D.** ILA concentration in tears. **C:** Control group (n=3), fitted with 0 D lenses and administered PBS topically to the ocular surface three times daily. **LIM:** Lens-induced myopia model group (n=3), fitted with -30 D lenses and administered PBS topically to the ocular surface three times daily. **LIM-T:** Lens-induced myopia treatment group (n=3), fitted with -30 D lenses and administered probiotic solution topically to the ocular surface three times daily. Data are presented as mean ± SD from three independent biological replicates (n = 3 mice per group). Statistical significance among the three groups was assessed by one‑way ANOVA followed by Tukey's post hoc test for multiple comparisons. All comparisons were pre‑planned and unpaired. **P* < 0.05, ***P* < 0.01, ****P* < 0.001. "ns" indicates no significant difference (*P* ≥ 0.05).

**Figure S10 AHR inhibition abrogates the protective effect of *Lactobacillus mucosae* MM-1 on HM progression in mice via disruption of TGF-β/Smad suppression. A**. Research group design flowchart. See symbol legend: **↓** (time points); (device with HM modeling); (termination/euthanasia). **B.** Weekly body weight monitoring in experimental mice. **C.** Post-experimental refractive error (D) (n=10). **D.** Post-experimental axial length (AL) (n=10). **E.** Differences in refractive error (D) (n=10). **F.** Differences in axial length (AL) (n=10). **G-O.** Relative mRNA expression level of scleral *Ahr*, *Cyp1a1*, *Hif1α*, *Tgfb1*, *Smad2*, *Smad3*, *Smad7*, *Acta2* (α-SMA), and *Col1a1* (n=3). **P-S.** Western blot analysis of HIF-1α, α-SMA, and Col1a1 expression (n=3). **T-X.** Western blot analysis of Ahr, CYP1A1, TGF-β1, and Smad7 expression (n=3). **Y-ZA.** Western blot analysis of pSmad2, Smad2, pSmad3 and Smad3 expression (n=3). **ZB.** HE staining of scleral tissue (×400; scale bar=20 μm). **M:** Model group (n=10), fitted with -30D lenses and administered 5 μl of 0.05% DMSO topically to the ocular surface three times daily. **LT:** *L. mucosae* MM-1 treatment group (n=10), fitted with -30 D lenses and administered probiotic solution topically to the ocular surface three times daily. **LTI:** *L. mucosae* MM-1 and inhibitor treatment group (n=10), fitted with -30D lenses and administered 5 μL of CH-223191 (10 μM) followed by 5 μL of probiotic solution (10^6^ CFU/mL), both applied topically to the ocular surface three times daily. Data are presented as mean ± standard deviation (SD) from independent biological replicates (n = 10 or 3 mice per group). Statistical significance was assessed as follows: paired data (left vs. right eyes) by two‑way ANOVA followed by Šidák's multiple comparisons test; unpaired data by one‑way ANOVA followed by Tukey's post hoc test. All comparisons were pre‑planned. **P* < 0.05, ***P* < 0.01, ****P* < 0.001. "ns" indicates no significant difference (*P* ≥ 0.05).

**Table S1. PCR primer sequences.**

| Gene | Direction | Primer sequence (5’-3’) | Product length (bp) |
| --- | --- | --- | --- |
| *Lactobacillus* | Forward | TGGAAACAGRTGCTAATACCG | / |
|  | Reverse | GTCCATTGTGGAAGATTCCC |  |
| 16SrRNA | Forward | ACTCCTACGGGAGGCAGCAGT | / |
|  | Reverse | TATTACCGCGGCTGCTGGC |  |
| *Col1a1* | Forward | TAAGGGTCCCCAATGGTGAGA | 203 |
|  | Reverse | GGGTCCCTCGACTCCTACAT |  |
| *Acta2* | Forward | GGCTGTTTTCCCATCCATCGTG | 148 |
|  | Reverse | GGTGATGATGCCGTGTTCTATCG |  |
| *Gapdh* | Forward | GAAGGTCGGTGTGAACGGAT | 168 |
|  | Reverse | ACTGTGCCGTTGAATTTGCC |  |
| *Tgfb1* | Forward | ACTGGAGTTGTACGGCAGTG | 123 |
|  | Reverse | GGGGCTGATCCCGTTGATTT |  |
| *Cyp1a1* | Forward | AAGGTGGTAGTTCTTGGAGCTT | 100 |
|  | Reverse | ATGATCTAGGTGGCTGCTTGG |  |
| *Ahr* | Forward | GCTTCTTTGATGGCGCTGAA | 213 |
|  | Reverse | GTCCACTCCTTGTGCAGAGT |  |
| *Smad2* | Forward | CCAAGCACTTGCTCTGAAATTTGG | 110 |
|  | Reverse | CGGCCATCAAGAGACCTGGTT |  |
| *Smad3* | Forward | CATCACCACGCAGAACGTGA | 257 |
|  | Reverse | CCAACACTGGAGGTAGAACTGG |  |
| *Smad7* | Forward | TCTCAAACCAACTGCAGGCT | 254 |
|  | Reverse | AATTGAGCTGTCCGAGGCAA |  |
| *Hif1a* | Forward | CCTGTAAGCAAGGAGCCAGA | 281 |
|  | Reverse | CTGTCTAGACCACCGGCATC |  |
| *HIF1A* | Forward | AGAGGTTGAGGGACGGAGAT | 175 |
|  | Reverse | GACGTTCAGAACTTATCCTACCAT |  |
| *ACTA2* | Forward | CTATGAGGGCTATGCCTTGCC | 122 |
|  | Reverse | GCTCAGCAGTAGTAACGAAGGA |  |
| *COL1A1* | Forward | GAGGGCCAAGACGAAGACATC | 140 |
|  | Reverse | CAGATCACGTCATCGCACAAC |  |
| *AHR* | Forward | TGGTTGTGATGCCAAAGGAAG | 217 |
|  | Reverse | AAGCAGGCGTGCATTAGACT |  |
| *SMAD2* | Forward | CCCAGCAGGAATTGAGCCA | 129 |
|  | Reverse | TCAAGAAGAGTGTCATCTGTGTCC |  |
| *CYP1A1* | Forward | TCGGCCACGGAGTTTCTTC | 141 |
|  | Reverse | GGTCAGCATGTGCCCAATCA |  |
| *TGFB1* | Forward | CACGTGGAGCTGTACCAGAA | 226 |
|  | Reverse | CGGTAGTGAACCCGTTGATG |  |
| *SMAD3* | Forward | CCATCTCCTACTACGAGCTGAA | 149 |
|  | Reverse | CACTGCTGCATTCCTGTTGAC |  |
| *SMAD7* | Forward | AAGATCAACCCCGAGCTGGT | 133 |
|  | Reverse | GCACAGCATCTGGACAGTCAGT |  |
| *AXIN2* | Forward | CAACACCAGGCGGAACGAA | 103 |
|  | Reverse | GCCCAATAAGGAGTGTAAGGACT |  |
| *CCND1* | Forward | ATGCCAACCTCCTCAACGAC | 159 |
|  | Reverse | TCTGTTCCTCGCAGACCTCCA |  |
| *YAP1* | Forward | TAGCCCTGCGTAGCCAGTTA | 177 |
|  | Reverse | TCATGCTTAGTCCACTGTCTGT |  |
| *CTGF* | Forward | CAGCATGGACGTTCGTCTG | 115 |
|  | Reverse | AACCACGGTTTGGTCCTTGG |  |
| *CYR61* | Forward | GTACAGCAGCCTGAAAAAGGG | 115 |
|  | Reverse | GCAGTACTTGGGCCGGTATT |  |
| *FOS* | Forward | GGGGCAAGGTGGAACAGTTAT | 126 |
|  | Reverse | CCGCTTGGAGTGTATCAGTCA |  |
| *JUN* | Forward | GCTGCTCTGGGAAGTGAGTT | 136 |
|  | Reverse | TTAAGATGCCTCCCGCACTC |  |
| *AKT1* | Forward | AGCGACGTGGCTATTGTGAAG | 96 |
|  | Reverse | GCCATCATTCTTGAGGAGGAAGT |  |
| *HES1* | Forward | TCAACACGACACCGGATAAAC | 153 |
|  | Reverse | GCCGCGAGCTATCTTTCTTCA |  |
| *HEY1* | Forward | CGGACGAGAATGGAAACTTGA | 183 |
|  | Reverse | GCTCCATTACCTGCTTCTCA |  |
| *GAPDH* | Forward | CTAGCTGGCCCGATTTCTCC | 271 |
|  | Reverse | GAGGGATCTCGCTCCTGGAA |  |

| Antibody | Target | Host Species | Manufacturer | Catalog No. | Dilution |
| --- | --- | --- | --- | --- | --- |
| Primary | α-SMA | Rabbit | Proteintech | 14395-1-AP | 1:2000-1:12000 |
| Primary | α-tubulin | Mouse | Proteintech | 66031-1-Ig | 1:20000-1:100000 |
| Primary | COL1A1 | Rabbit | ABclonal | A24112 | 1:1000-1:2000 |
| Primary | pSmad2 | Rabbit | Selleck | F1689 | 1:1000 |
| Primary | Smad2 | Mouse | Abcam | ab119907 | 1:500-1:2000 |
| Primary | TGF-β1 | Rabbit | Abcam | ab215715 | 1:1000 |
| Primary | AHR | Rabbit | Proteintech | 28727-1-AP | 1:1000-1:3000 |
| Primary | Smad3 | Mouse | Proteintech | 66516-1-Ig | 1:2000-1:12000 |
| Primary | pSmad3 (S423 + S425) | Rabbit | Abmart | T55140 | 1:500-1:2000 |
| Primary | HIF-1α | Rabbit | Abcam | ab179483 | 1:1000 |
| Primary | CYP1A1 | Rabbit | Proteintech | 13241-1-AP | 1:1000-1:4000 |
| Primary | Smad7 | Rabbit | Proteintech | 25840-1-AP | 1:500-1:3000 |
| Secondary | Anti-rabbit IgG | Goat | Proteintech | SA00001-2 | 1:2000 |
| Secondary | Anti-mouse IgG | Goat | Proteintech | SA00001-1 | 1:2000 |

**Table S2 Antibodies used in this study**
